# Molecular determinants of neuropeptide-mediated activation mechanisms in tachykinin NK1 and NK2 receptors

**DOI:** 10.1016/j.jbc.2024.107948

**Published:** 2024-10-30

**Authors:** Jacob E. Petersen, Artem Pavlovskyi, Jesper J. Madsen, Thue W. Schwartz, Thomas M. Frimurer, Ole H. Olsen

**Affiliations:** 1 Section for Metabolic Receptology, Novo Nordisk Foundation Center for Basic Metabolic Research, University of Copenhagen, Copenhagen, Denmark; 2 Department of Molecular Medicine, Morsani College of Medicine, University of South Florida, Tampa, Florida, USA; 3 Center for Global Health and Infectious Diseases Research, Global and Planetary Health, College of Public Health, University of South Florida, Tampa, Florida, USA

**Keywords:** G protein–coupled receptor (GPCR), mutagenesis, signaling, peptide interaction, molecular dynamics, molecular modeling

## Abstract

Substance P and neurokinin A are closely related neuropeptides belonging to the tachykinin family. Their receptors are neurokinin one receptor (NK1R) and neurokinin two receptor (NK2R), G protein–coupled receptors that transmit G_s_ and G_q_-mediated downstream signaling. We investigate the importance of sequence differences at the bottom of the receptor orthosteric site for activity and selectivity, focusing on residues that closely interact with the C-terminal methionine of the peptide ligands. We identify a conserved serine (NK1R-S297^7.45^) and the position of the tryptophan residue within the canonical “toggle switch” motif, CWxP of TM6, neighboring a phenylalanine in NK1R (NK1R-F264^6.51^) and a tyrosine in NK2R (NK2R-Y266^6.51^), giving rise to distinct microenvironments for the neuropeptide C terminals. Mutating these residues results in dramatic activity changes in both NK1R and NK2R due to a close interaction between the ligand and toggle switch. Structural analysis of active and inactive NKR structures suggests only a minor change in sidechain rotation of toggle switch residues upon activation. However, extensive molecular dynamics simulations of receptor:neuropeptide:G protein complexes indicate that a major, concerted motion happens in the toggle switch tryptophan indole group and the sidechains of the microswitch motif Pro-Ile-Phe (PIF). This rotation establishes a tight hydrogen bond interaction from the tryptophan indole to the conserved serine (NK1R-S297^7.45^) and a mainchain carbonyl (NK1R-A294^7.41^) in the kink of TM7. This interaction facilitates communication with the NPxxY microswitch motif of TM7, resulting in stabilization of the G protein–binding region. NK1R-S297^7.45^ is consequently identified as a central hub for the activation of NKRs.

Extensive investigations have been conducted over the years in the pursuit of understanding the neuropeptide-dependent signaling system of the neurokinin receptor (NKR) family (1, 2). There are three NKRs, NK1R, NK2R, and NK3R, each with its own natural high-affinity neuropeptide ligand, substance P (SP), neurokinin A (NKA), and neurokinin B (NKB), respectively. These peptides share a conserved C-terminal sequence motif known to elicit activity toward any of the three NKRs but exhibit sequence variation toward the N terminus (1). NKRs represent attractive drug targets, which underscores the need for detailed structural and functional information on NKRs and their activation mechanisms. NKRs have been implicated in eye disorders (3), chemotherapy-induced nausea and vomiting through approved NK1R antagonists, treatment of anxiety disorders and asthma (4, 5, 6) through NK2R antagonists, target for conditions such as schizophrenia, hypertension, reproductive disorders, and preeclampsia *via* NK3R (7), and treatment of obesity and obesity-related issues through several of the NKRs (8, 9, 10, 11, 12).

Despite sharing the common C-terminal sequence, the neuropeptide agonists exhibit some degree of cross-reactivity toward their nonnatural receptors (1). We recently demonstrated that NK1R and NK2R specificity and cross-reactivity toward their peptide ligands can be explained by the interactions between the amino acids preceding the amidated FxGLM consensus motif of the bound peptide ligand and two regions of the receptor: the β-hairpin of the extracellular loop (ECL) two and a N-terminal segment leading into transmembrane (TM) helix 1 (13). For NK3R, it has been shown that the specific (14) interactions of the N-terminal of agonists NKB, SP as well as a peptide analog, senktide, to the N-terminal region of NK3R, ECL2, and ECL3 determine specificity and potency (7). However, certain details, including the lack of constitutive activity in unstimulated NKRs are a poorly understood characteristic (15, 16, 17). Biased signaling in G protein–coupled receptors (GPCRs) refers to the selective activation of specific intracellular signaling pathways by ligands, resulting in distinct functional outcomes, and the concept has significant importance in drug development (18, 19, 20).

Structural information on the NKRs has expanded rapidly in recent years with X-ray crystallographic structures of NK1R in complex with a variety of antagonists recently being reported (21, 22, 23), opening the avenue for ensemble exploration (24, 25, 26) and novel functional insights. Furthermore, structures of NK1R in complex with SP and signal mediators, G_q_ and G_s_, NKA-bound NK2R in complex with the signal mediator G_q_, and NK3R in complex SP, NKB, senktide, and signal mediator G_q_ were solved by cryo-EM (6, 7, 27, 28). Notably, in the cryo-EM structures of NK1R bound to SP with either G_q_ or G_s_, both G proteins exhibit almost identical conformations in relation to NK1R. The sole subtle difference is that the Cα5 helix of G_q_ is positioned slightly deeper within NK1R than G_s_ (27), suggesting minimal or no biased signaling. Nonetheless, several studies have reported biased signaling in NK1R (15, 28). Analysis of antagonist-bound complexes in an inactive state and active agonist-bound complexes bound to G proteins for NK1R reveal the primary rearrangements of the TM helices during activation (Fig. 1, *A* and *B*). The binding of SP to NK1R induces extracellular contraction of the orthosteric pocket, characterized by an inward motion of TM helices 6 and 7, along with ECL3 as illustrated in Fig. 1*A*. Additionally, there is a substantial intracellular outward movement of TM6 and minor rearrangements in TM3 and TM4, as depicted in Fig. 1*B*. These rearrangements reposition F264^6.51^ (Ballesteros–Weinstein numbering (29)) at the bottom of the pocket to trigger the toggle switch W261^6.48^ (being part of the microswitch motif CWxP (30)), which is placed one helix turn right below F264^6.51^, Fig. 1, *C*–*E*. Consequently, these changes lead to an outward shift of F257^6.44^, a constituent of the PIF microswitch motif. The toggle switch is sandwiched between the two aromatic rings, undergoing a reorientation and restriction. The outcomes of these relatively subtle alterations illuminated by crystal and cryo-EM structures, which are supposed to manifest in the formation of an intracellular cavity conducive to G protein binding (27), motivates our current investigation into the importance of F264^6.51^. Interestingly, more than a decade ago, the toggle switch W261^6.48^ and F257^6.44^ (31, 32), were examined by mutagenesis and molecular dynamics (MD) simulations and concluded to be part of the activation mechanism. Sequence alignment of NKRs (Fig. S1) shows that the F264^6.51^ is replaced by tyrosine in NK2R and NK3R. In cryo-EM structures of NK2R and NK3R, the tyrosine hydroxyl group has the potential to form a hydrogen bond (H-bond) to the amide of the C-terminal methionine of NKA (Fig. 1*F*) and NKB, respectively, thereby potentially stabilizing the toggle switch in an active conformation in NK2R and NK3R more so than would be the case in NK1R. Another notable sidechain located near F264^6.51^ distinct from NK1R at the bottom of the orthosteric site is M291^7.38^. NK2R and NK3R have a phenylalanine residue at the corresponding position (shown for NK2R in Fig. 1*F*). In Fig. 1*G*, the interaction between M291^7.38^ in NK1R (depicted in gray stick model) to F264^6.51^ is illustrated. Mutating 291 to phenylalanine in NK1R may therefore stabilize the toggle switch further *via* an aromatic ring stacking interaction to its neighbor F264^6.51^. A third sidechain at the bottom of the orthosteric site, near the agonist’s C-terminal methionine, is the valine at position 116 of NK1R, which attracted our interest as well. The corresponding sidechain in NK2R is a methionine.

**Figure 1:**
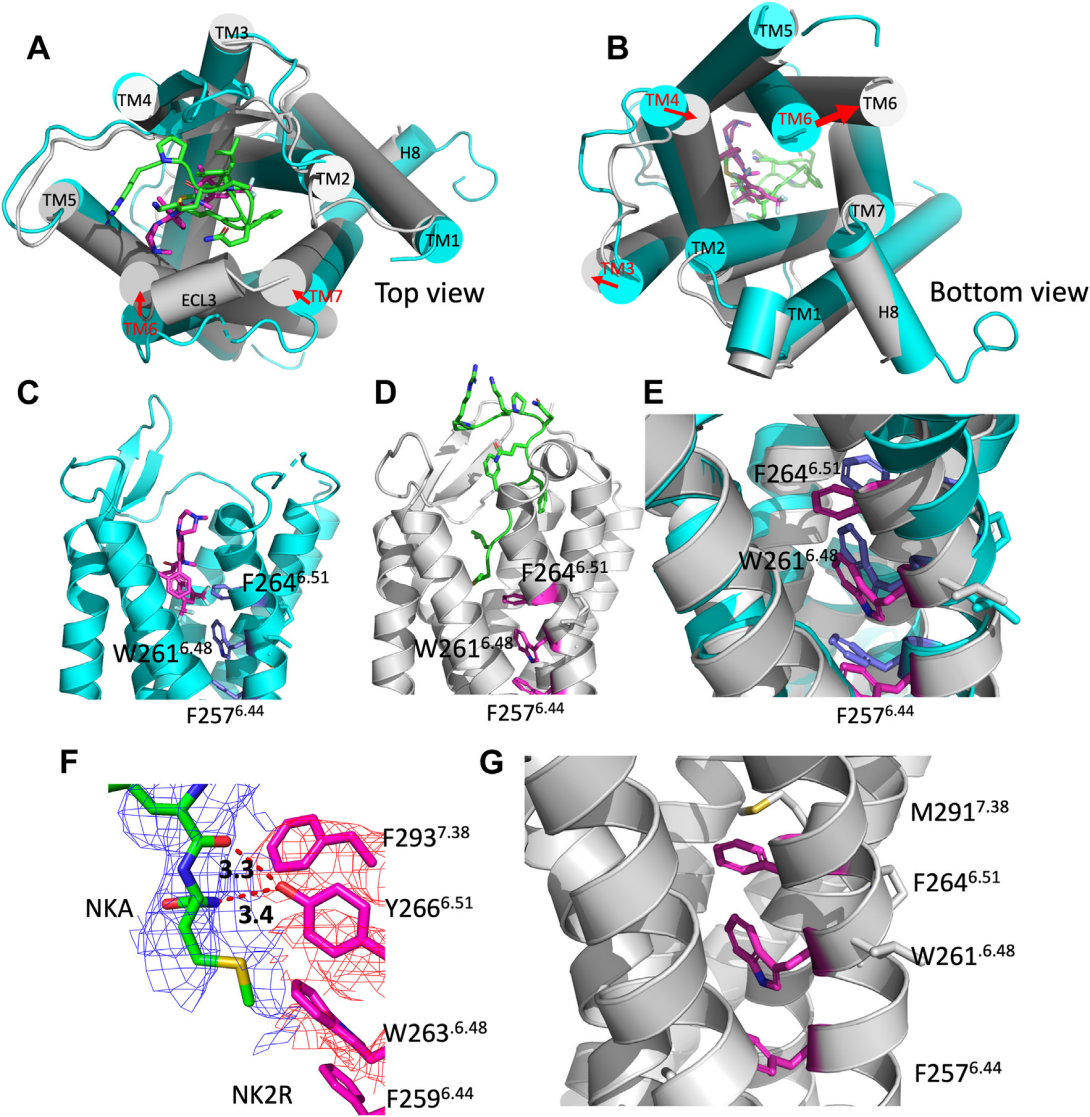
**Activation of NK1R.***A*, (*top* (extracellular) *view*) and *B*, (*bottom* (intracellular) *view*), primary rearrangements of TM helices (TM 1–7), shown as *cylinders*, during activation. Antagonist (*magenta*) and SP (*green*) in stick models. Inactive antagonist-bound structure (PDB ID: 6hlp) in *cyan* and active agonist-bound structure (PDB ID: 7p00) in *gray*. Major shifts in TM helix orientations are marked by *red arrows*: TM helices 6 and 7 in the extracellular region and TM 3, TM 4, and TM 6 in the intracellular region. *C* and *D*, NK1R in *cartoon*, side view of inactive (active) structure, residues from the microswitch motifs CWxP and PIF, and F264^6.51^ in *blue* (*magenta*). *E*, overlay of inactive and active structures (ligands omitted). The toggle switch W261^6.48^ is sandwiched between F257^6.44^ and F264^6.51^. TM6 reorientates upon activation, thereby opening the intracellular cavity. *F*, the interaction between NKA (in *green stick model*, *blue cryo-EM density mesh*) and NK2R-Y266^6.51^ (in *magenta stick model, red cryo-EM density mesh*) observed in the AF2 structure prediction of NK2R:NKA (13). H-bonds involving the hydroxyl group of NK2R-Y266^6.51^ are highlighted by *red dashed lines*. Mutants of NK1R incorporating F264^6.51^Y and M291^7.38^F are anticipated to exhibit a comparable arrangement. *G*, the close proximity of M291^7.38^ in NK1R (depicted in *gray stick model*) to F264^6.51^ is significant, suggesting that the mutation of M291^7.38^ to phenylalanine could stabilize the toggle switch W261^6.48^ through an aromatic interaction with F264^6.51^. NKA, neurokinin A; NKR, neurokinin receptor; SP, substance P; TM, transmembrane.

Considering the renewed interest in specific and selective agonists to NKRs, we set out to examine the impact of these binding pocket amino acid positions and their variation among the NKRs might have on modulation of microswitches and resultant receptor activation. In particular, the following conserved mutations were introduced in NK1R: F264^6.51^Y and M291^7.38^F (and their combination) and likewise NK2R were mutated to the residues corresponding to those in NK1R: Y266^6.51^F and F293^7.38^M (and their combination). Further, the impact of changes in position 116 of NK1R and the corresponding position in NK2R was examined. Consequently, the mutations NK1R, V116^3.36^M and NK2R: M117^3.36^V, were introduced and their combinations with the previous mutations discussed were analyzed. All constructs were subjected to interrogation by MD simulations, bioluminescence resonance energy transfer (BRET)-based cAMP, IP_3_ accumulation, and β-arrestin recruitment assays to elucidate the role of these residues as molecular determinants in activation and signaling.

However, introducing changes at position 116 of NK1R and the corresponding position in NK2R showed no impact. Therefore, these changes are not discussed further.

The paper is outlined as follows. First, the impact of the two sets of mutants in NK1R and NK2R is undertaken. Dramatic changes in activities are observed for mutations introduced in both NK1R and NK2R. Hence, the activation is particularly impacted for mutants NK1R-F264^6.51^Y and NK2R-Y266^6.51^F. The former exhibits dramatic activity increase (both in potency and efficacy) when activated by NKA and only minor improvement in efficacy when activated by SP, while the latter loses activity (both in potency and efficacy) when activated by NKA and completely lacks activity when activated by SP. The combination of these mutants with NK1R-M291^7.38^F and NK2R-F293^7.38^M enhances the observed activity changes.

Second, the impact of motif swaps at a single amino acid position near the toggle switch necessitated an extensive MD simulation study to investigate the behavior of the surrounding environment. The outcome suggests that after binding of the endogenous ligand in the NK1R:SP complex, the toggle switch takes part in a concerted rotation with the sidechains of the microswitch motif PIF resulting in an H-bond to the sidechain of NK1R-S297^7.45^. Further, in simulations of the complex NK1R:SP:G_q_, the indole group of the toggle switch tryptophan establishes one additional H-bond to the mainchain carbonyl of NK1R-A294^7.41^. This facilitates the maturation of the interaction region to the G proteins. To investigate the impact of mutations at position NK1R-297^7.45^ on receptor activity, we examined the previously reported mutation NK1R-S297^7.45^A, which demonstrated a significant reduction in activity (15). MD simulations revealed that this mutant fails to establish hydrogen bonds from the toggle switch tryptophan to the two H-bond acceptors. The same observation was also noted for the NK1R-S297^7.45^N mutation analyzed in the present study.

## Results and discussions

### Activation of NK1R and NK2R orthosteric site-mutants by SP and NKA

Structural comparison (Fig. 1) and sequence alignment (Fig. S1) revealed regions at the bottom of the orthosteric sites of NK1R and NK2R that affect agonist binding and signaling through interactions with the toggle switch residue NK1R-W261^6.48^ of the microswitch motif CWxP (Fig. 1, *C*–*E*) (15). The pivotal role of the conserved toggle switch residues has been elucidated by mutagenesis (33, 34) Hence, the mutation NK1R-W261^6.48^A exhibits no activity after stimulation with SP (15). One helix-turn above the toggle switch, the mutated NK1R-F264^6.51^Y can potentially form an H-bond with the amide of the C-terminal SP methionine. If mutated to phenylalanine, the adjacent M291^7.38^ could therefore stabilize the aromatic ring at position 264^6.51^ *via* an aromatic π–π stacking interaction (Fig. 1, *F* and *G*). Based on these considerations, we introduced mutations F264^6.51^Y and M291^7.38^F (and their double mutant combination) in NK1R, corresponding to the residues in NK2R. Conversely, mutations Y266^6.51^F and F293^7.38^M (and their double mutant combination) were introduced in NK2R. These mutants were assessed in BRET-based cAMP, IP_3_ accumulation, and β-arrestin recruitment assays. The BRET-based cAMP assay is expected to reflect G_s_-dependent signaling, while IP_3_ accumulation assay is anticipated to reflect G_q_-dependent signaling (15, 35).

#### NK1R-F264^6.51^Y mutant adds direct H-bond with bound SP

**Figure 2:**
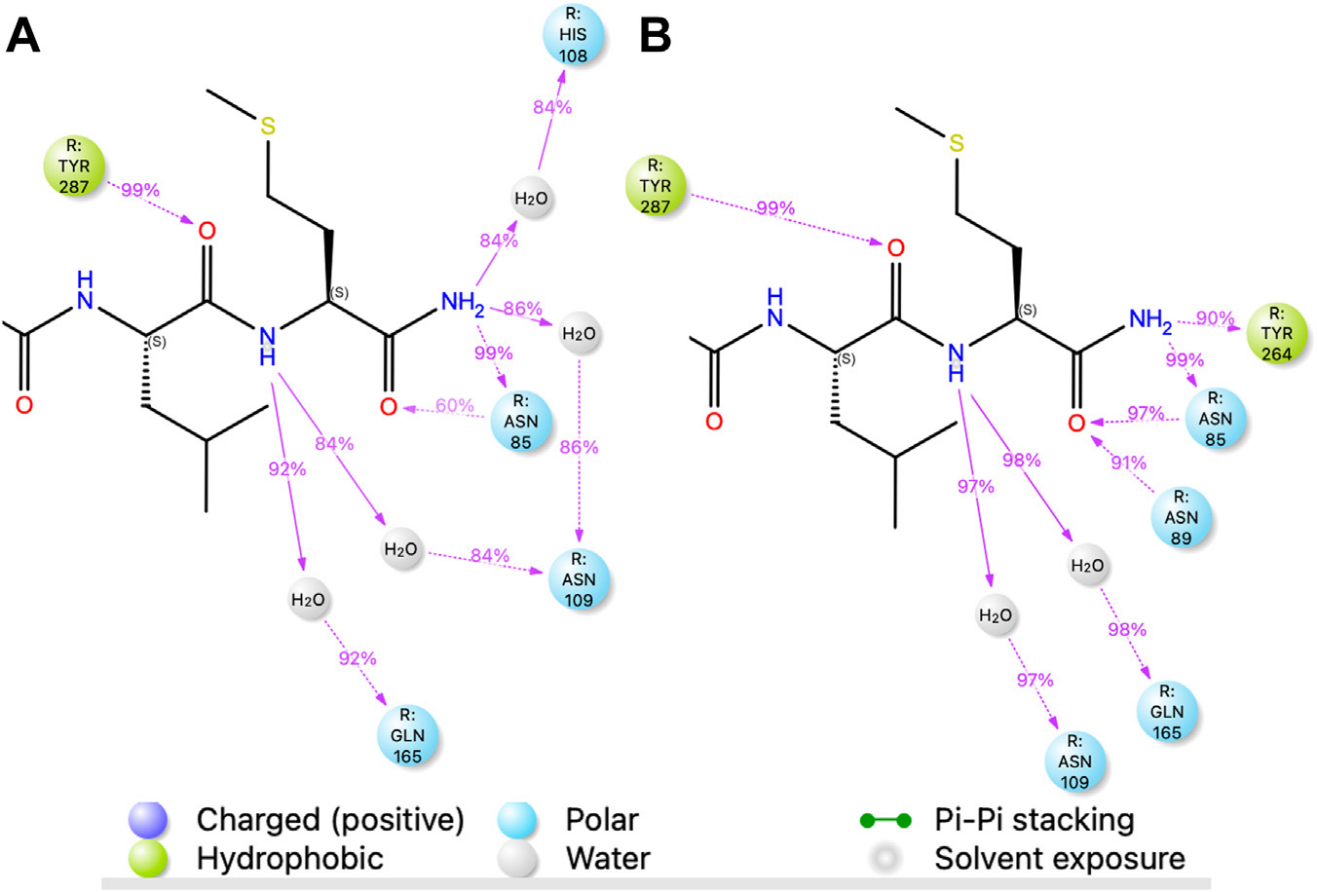
**Interaction of SP10-11 (LM-NH_2_) with NK1R and NK1R-F264^6.51^Y**. Interaction diagrams generated from MD trajectories (1500 ns). Features appearing in the diagram and what they represent are shown below the diagrams. Interactions between the C-terminal amide of SP are different for the two receptors. *A, f*or NK1R two waters mediate the H-bonds between C-terminal amide NH_2_ and NK1R-N109^3.29^/NK1R-H108^3.28^. *B*, in NK1R-F264^6.51^Y, the water molecules are missing and a direct H-bond is established between F264^6.51^Y and the amide. MD, molecular dynamics; NKR, neurokinin receptor; SP, substance P.

MD simulations were performed on the NK1R:SP and NK1R-F264^6.51^Y:SP complexes to evaluate the impact of this mutation. The analysis of the trajectories is illustrated in simulation interaction diagrams, showing the frequency or persistence of interaction strength throughout the simulation. This highlights which interactions are stable and which are more transient. Fig. 2 displays contact diagrams illustrating the interactions between SP10-11 and NK1R (Fig. 2*A*) and NK1R-F264^6.51^Y (Fig. 2*B*). The interactions between SP1-9 and the two receptor constructs are identical, as shown in Fig. S2. This observation indicates that there is no communication between the ligand termini. Hence, mutations close to the ligand C terminus have no impact on N-terminal binding. Interestingly, interactions involving the C-terminal amide of SP differ between the two receptors. For NK1R, two water molecules mediate the H-bonds between C-terminal amide NH_2_ and NK1R-N109^3.29^/NK1R-H108^3.28^ (Fig. 2*A*). In NK1R-F264^6.51^Y, the water molecules are displaced, allowing a direct H-bond to form between F264^6.51^Y and the amide (Fig. 2*B*). This observation suggests an increased affinity of SP for NK1R-F264^6.51^Y due to an entropic effect (36). Consequently, it is expected that the NK2R-Y266^6.51^F mutant will exhibit the opposite effect, likely lacking a direct H-bond interaction and resulting in decreased binding affinity. Based on the simulations, we anticipate that the introduction of mutations will affect the binding affinity.

#### Probing G_s_ and G_q_ activation, and β-arrestin recruitment in NK1R mutants NK1R-F264^6.51^Y and NK1R-M291^7.38^F

**Figure 3:**
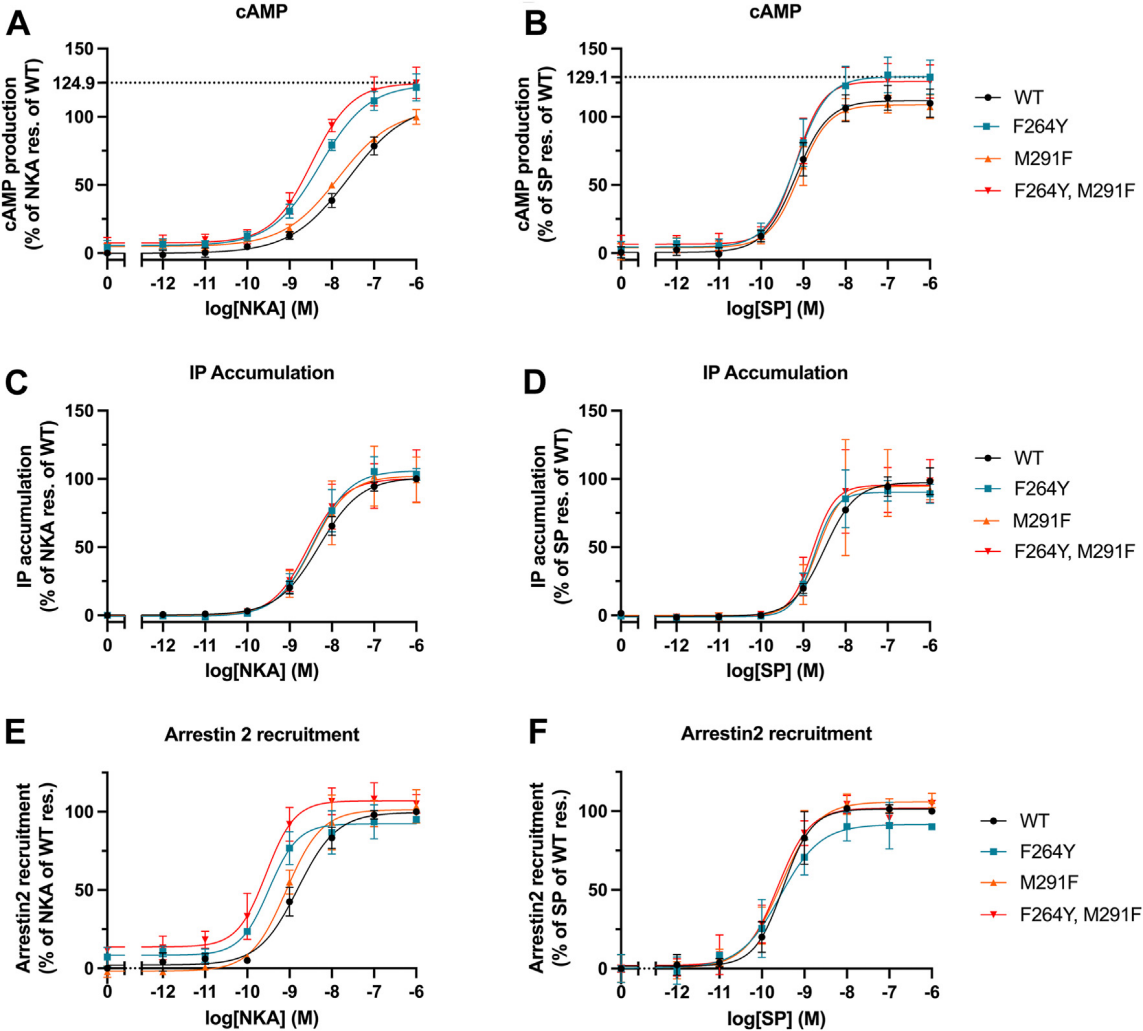
**Activation of NK1R mutants.***A*-*F*, NK1R WT (*black*), NK1R F264Y (*blue*), NK1R M291F (*orange*) and NK1R F264Y, M291F (*red*). BRET-based cAMP assay (G_s_): NK1R mutants activated by NKA (*A*) and SP (*B*). *A*, NKA effectively activates NK1R mutants, particularly single mutant NK1R-F264^6.51^Y impacts both potency and efficacy. *B*, SP exhibits only a weak increase in activation (efficacy) of NK1R mutants. IP_3_ accumulation assay (G_q_): *C* and *D*, NK1R mutants are activated by NKA and SP. A minor increase in NKA activation of mutants. No impact of SP on activation of NK1R mutants. In Table S1*A* the EC_50_ and E_max_ values from functional assays corresponding to panels (*A*–*D*) are tabulated. β-arrestin recruitment: (*E* and *F*) Functional consequence of NK1R mutants on β-arrestin mobilization. In panel (*E*), the influence of mutations activated by NKA is similar to the effects observed in the BRET-based cAMP assay (G_s_). *F*, the mutants activated by SP show minor difference in β-arrestin mobilization. All data are shown as mean ± SEM of three independent experiments, each run with technical triplicates. BRET, bioluminescence resonance energy transfer; NKA, neurokinin A; NKR, neurokinin receptor; SP, substance P.

Figure 3, *A* and *B* present the results of the BRET-based cAMP assay. The upper panels highlight NK1R mutants activated by NKA and SP, respectively. Activation by NKA shows a significant increase in both potency and efficacy, especially for NK1R-F264^6.51^Y, with a 5-fold enhancement in potency and a 25% increase in efficacy compared to NK1R. It is of note that the expression levels for mutants containing F264^6.51^Y are approximately 50% (Fig. S3) of that of NK1R which may point to an even larger impact. This enhancement is further amplified when combined with NK1R-M291^7.38^F in the double mutant, resulting in a total potency improvement of 7.5-fold. The potency approaches that observed for NK1R activated by SP, with all mutants demonstrating improved efficacy compared to NK1R activated by NKA. The impact of the NK1R mutations on activation by SP is less pronounced, though enhancements in efficacy are still evident.

The expected formation of a direct H-bond due to the NK1R-F264^6.51^Y mutation significantly influences NKA activation, while changes in SP activation are comparatively modest. This observation underscores the importance of the two mutated residues at the bottom of the orthosteric site in drug design processes, where selectivity is crucially important.

Figure 3, *C* and *D* show the outcomes of the IP_3_ accumulation assay, revealing different results from the BRET-based cAMP assay. Here, the mutants exhibit only marginal improvements in activity for both NKA and SP activation compared to NK1R activation, indicating a distinct mode of activation for G_s_ and G_q_-dependent activation. Therefore, the effects of the proposed formation of a direct hydrogen bond, which leads to stabilization and enhanced binding, is only evident in the BRET-based cAMP assay, suggesting a signaling bias toward G_s_.

Figure 3, *E* and *F* show results from the β-arrestin recruitment assay. In panel E, the impact of mutations activated by NKA mirrors the effects seen in the BRET-based cAMP assay (Gs, panel A), with a consistent ranking in potency. In panel F, the mutants activated by SP show minor difference in β-arrestin mobilization. The potencies of NK1R mutations activated by SP are similar in all three assays (panels B, D, and F). Interestingly, activation by SP of a set of NK1R Ala substitutions of residues participating in a water H-bond network (15) demonstrated that the β-arrestin mobilization effects were similar to those observed in IP_3_ accumulation assay (G_q_).

#### Probing G_s_ and G_q_ activation, and β-arrestin recruitment in NK2R mutants Y266^6.51^F and F293^7.38^M

**Figure 4:**
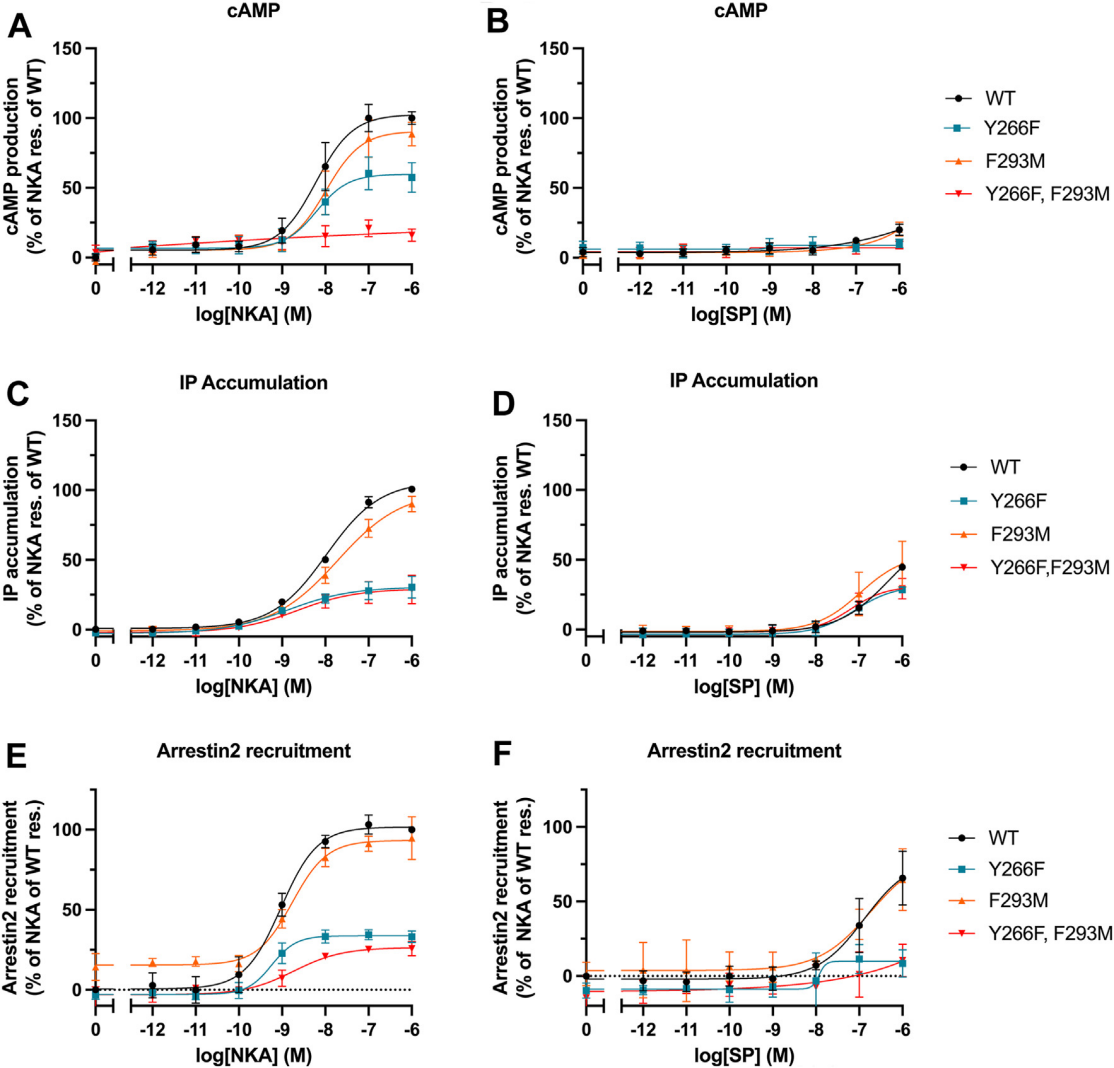
**Activation of NK2R mutants.***A-F*, NK2R WT (*black*), NK2R F264Y (*blue*), NK2R M291F (*orange*), and NK2R F264Y, M291F (*red*). BRET-based cAMP assay (G_s_): NK2R mutants activated by NKA (*A*) and SP (*B*). *A*, NK2R mutants show a dramatic loss of activity. *B*, SP exhibits no activity. IP_3_ accumulation assay (G_q_): (*C* and *D*) NK2R mutants are activated by NKA (*C*) and SP (*D*) resulting in dramatic loss of activity as compared to WT NKRs as seen for the cAMP assay. In Table S1*B* the EC_50_ and E_max_ values from functional assays corresponding to panels (*A*–*D*) are tabulated. β-arrestin recruitment: (*E* and *F*) Functional consequence of NK1R mutants on β-arrestin mobilization. *E*, the influence of mutations activated by NKA is similar to the effects observed in both BRET-based cAMP assay (G_s_) and IP_3_ accumulation assay (G_q_). *F*, the mutants activated by SP show drastic reduction in β-arrestin mobilization. All data are shown as mean ± SEM of three independent experiments, each run with technical triplicates. BRET, bioluminescence resonance energy transfer; NKA, neurokinin A; NKR, neurokinin receptor; SP, substance P.

The data for NK2R mutants activated by NKA and SP in the BRET-based cAMP assay are shown in the upper panels of Fig. 4. The observed effect is contrary to that seen in the NK1R mutants (Fig. 3, *A* and *B*). The NK2R-Y266^6.51^F mutant demonstrates a significant decrease in both potency and efficacy when activated by NKA. Combining this mutant with NK2R-F293^7.38^M results in a mutant with almost no activity. When stimulated by SP, NK2R and all NK2R mutants completely lack measurable activity (Fig. 4*B*).

Figure 4, *C* and *D* illustrate the results of the IP_3_ accumulation assay for NK2R mutants. The responses to NKA and SP activation are consistent with those observed in the BRET-based cAMP assay (Fig. 4, *A* and *B*). As a result, the NK2R-Y266^6.51^F mutant exhibits a notable reduction in both potency and efficacy upon activation by NKA (Fig. 4*C*) and SP (Fig. 4*D*). Similar behavior is observed for the double mutant NK2R-Y266^6.51^F/F293^7.38^M. This suggests that the mutants activate the G proteins, albeit to a lesser extent.

Figure 4, *E* and *F* show results from the β-arrestin recruitment assay. In panel E, the influence of mutations activated by NKA is similar to the effects observed in both BRET-based cAMP assay (panel A, G_s_) and IP_3_ accumulation assay (panel B, G_q_). In panel F, the mutants activated by SP show drastic reduction in β-arrestin mobilization and a different response compared NK2R activated by NKA.

### Activation mechanism elucidated by all-atom MD simulations

**Figure 5:**
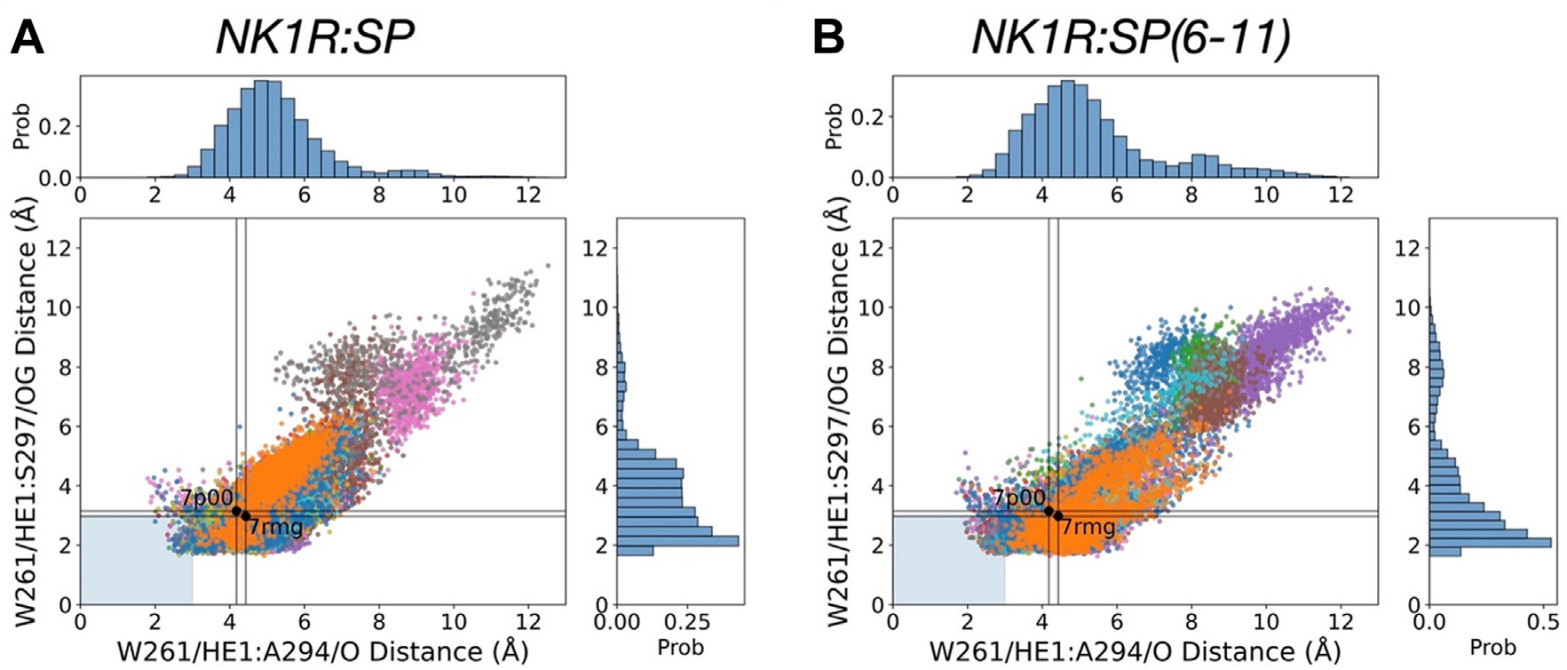
**Analyses of simulations of NK1R:SP and NK1R:SP(6–11) complexes.** The distances between indole of NK1R-W261^6.48^, the NK1R-A294^7.41^ mainchain carbonyl, and sidechain of NK1R-S297^7.45^ have been calculated and plotted against each other. Propensity histograms have been depicted. Twelve trajectories of each complex taken from (28) have been overlayed (in different colors). *A*, NK1R:SP and (*B*) NK1R:SP(6–11). *Black dots* indicate the distances in the cryo-EM structures. Large variations are observed but the results from the two complexes are very similar. From histograms of NK1R-W261^6.48^/HE1-NK1R-A294^7.41^/O distance, the absence of H-bond is apparent in contrast to the distance NK1R-W261^6.48^/HE1-NK1R-A297^7.45^/OG, where the histogram shows a high propensity for the presence of an H-bond. CHARMM atom types are used for reference. NKR, neurokinin receptor; SP, substance P.

The unexpected influence of conservative motif swaps at a single amino acid position around the toggle switch residue NK1R-W261^6.48^ described above prompted further investigation. It has been speculated that the activation mechanism is facilitated by minor structural changes in the toggle switch and its adjacent sidechains (Fig. 1) (27). To explore this notion, we conducted extensive MD simulations of NK1R in complex with SP and G_q_ starting from a cryo-EM structure (PDB ID: 7p00, (27)). Given the cryo-EM structures of NK1R bound to SP with either G_q_ or G_s_, both G proteins show nearly identical conformations in relation to NK1R (27), we will focus only on NK1R’s interaction with G_q_ in the following discussion. Interestingly, cryo-EM structures of NK1R bound to truncated SPs revealed a receptor-G protein conformation that is highly similar to the one observed with full-length SP (28). Examination of the cryo-EM density maps reveals a clearly defined orientation for the sidechain of NK1R-W261^6.48^. Maintaining this conformational consistency was initially regarded as a pivotal aspect of ensuring the robustness of the simulation. Surprisingly, it was observed that NK1R-W261^6.48^ and the surrounding sidechains consistently underwent large conformational changes after some time of simulation, deviating from the cryo-EM structure conformations. Such observations will invite the question of the influence of simulation details on the results. Therefore, a second, independent set of MD simulations were performed using another MD program (Desmond (37) instead of Gromacs (38)) and an entirely different forcefield (Optimized Potentials for Liquid Solutions (OPLS) (39) instead of CHARMM36m (40)), and the same sidechain reorientations were again observed. Similar simulations of a cryo-EM model of NK2R:NKA:G_q_ (13) yielded identical results. In MD simulations of NK1R:SP and NK2R:NKA complexes, identical conformational changes were again observed, although they occurred at different time points. Harris *et al.* (28) performed extensive MD simulations using yet another MD program (AMBER18 (2018), “Amber” https://sciencegateways.org/resources/amber), force field: CHARMM36m (40)) on the NK1R:SP and NK1R:SP(6–11) complexes (PDB ID: 7rmh (28)) to explore NK1R’s activation by SP and the shortened analog SP(6–11), which is probably more loosely bound to the receptor (28) but could share a similar mode of binding (14). Each complex was subjected to 12 independent 2 μs simulations (28). In Fig. 5, results from our analyses of the Harris *et al.* (28) trajectories are presented. Here, the toggle-switch NK1R-W261^6.48^ behaves similarly to the observations from MD simulations of NK1R:SP and NK2R:NKA complexes in the present study, differing from the conformation depicted in the cryo-EM structure, which will be discussed in more detail below. These findings suggest that the results from MD simulations warrant consideration and further analysis.

#### Activation mechanism

**Figure 6:**
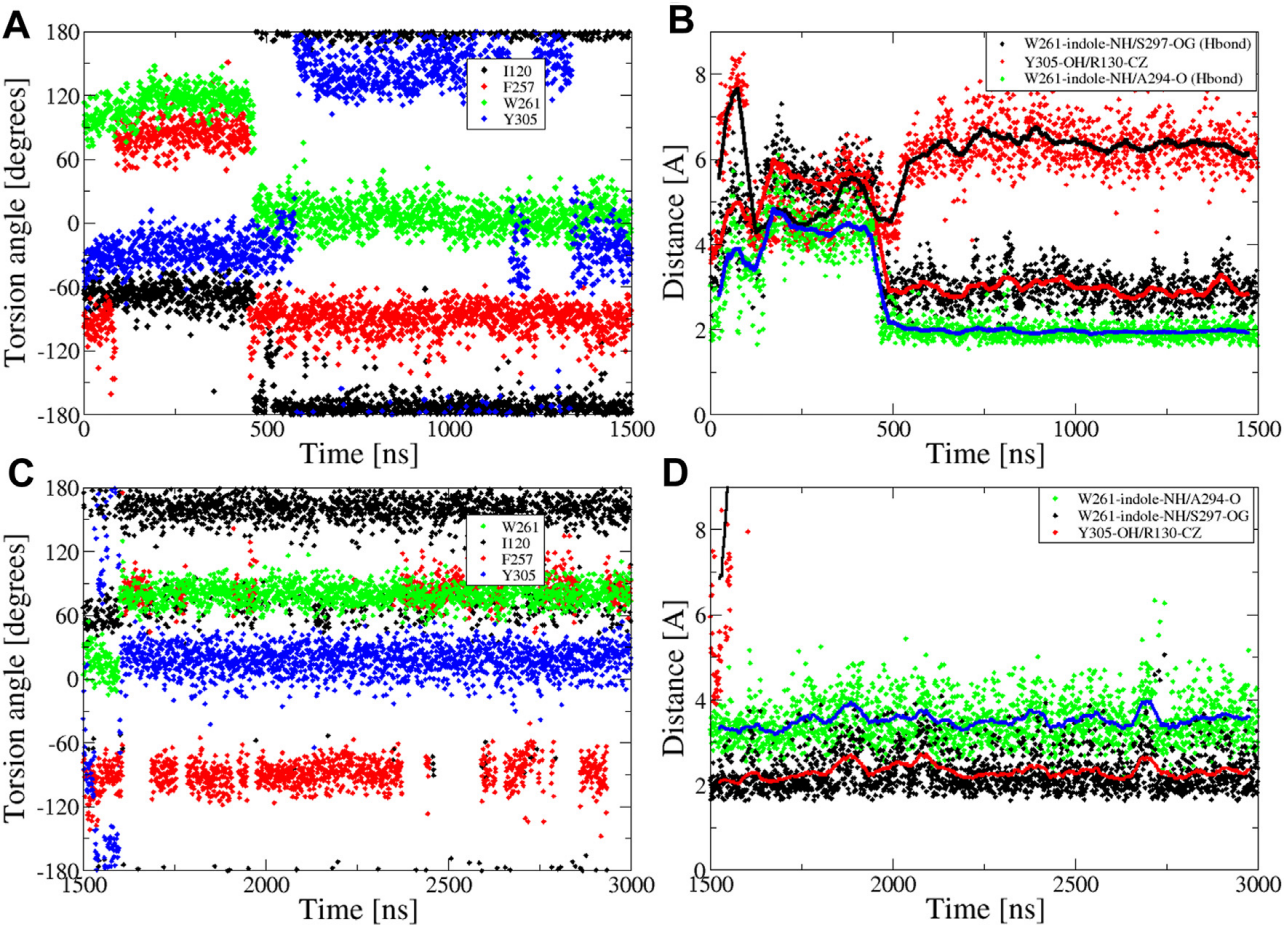
**MD simulations of NK1R:SP:G**_**q**_**complex and apo NK1R structure.***A*, concerted torsion rotation at 500 ns, χ_2_ torsion angles as a function of time for selected sidechains of microswitch motifs: NK1R-I120^3.40^ and NK1R-F257^6.44^ (PIF motif, *red*), NK1R-W261^6.48^ (C**W**xP motif, *green*), and NK1R-Y305^7.53^ (NPxx**Y**, *black*). The change of the latter motif is delayed compared to the other two. *B*, the initiation of H-bonds (after 500 ns) between the indole group of NK1R-W261^6.48^ and the sidechain of NK1R-S297^7.45^ (depicted in *black*) as well as to the mainchain carbonyl of NK1R-A294^7.41^ (depicted in *green*). The distance between sidechains of NK1R-R130^3.50^ (of the D**R**Y microswitch motif) and NK1R-Y305^7.53^ (of the NPxx**Y** microswitch motif) in *red*. *C* and *D*, simulation (of 1500 ns) of apo NK1R, where initial structure originates from last structure at time 1500 ns in (*A*–*B*) with SP and G_q_ removed and prepared according to protocol in Experimental procedures. *C*, the χ_2_ torsion angles for sidechains (with same coloring) as in (*A*). χ_2_ torsion angles shift after 100 ns. χ_2_ torsion angle for NK1R-W261^6.48^ (in *green*) returns to that observed for the first 500 ns in (*A*). *D*, distances as in (*B*), the H-bond pattern characteristic for the fully activated state disappears. Running averages have been shown to guide the eyes. CHARMM atom types are used for reference. NKR, neurokinin receptor; SP, substance P.

In Fig. 6*A*, the results of an MD trajectory of the NK1R:SP:G_q_ complex are shown, focusing on the torsion angles for selected sidechains within the microswitch motifs. These include NK1R-I120^3.40^ and NK1R-F257^6.44^ (PIF motif, red), NK1R-W261^6.48^ (CWxP motif, green) and NK1R-Y305^7.53^ (NPxxY motif, black). After approximately 500 ns, a concerted rotation of the three sidechains is observed. Rotation of NK1R-Y305^7.53^, located near the G_q_ binding region, is delayed by about 100 ns. Notably, the rotation of the toggle switch tryptophan enables the formation of two H-bonds from its indole group to the sidechain of NK1R-S297^7.45^ and the mainchain carbonyl of NK1R-A294^7.41^. These two residues reside in a kink of TM7, and the interaction is likely involved in transmitting information to the G_q_ binding region. This is further illustrated in Fig. 6*B*, which depicts the H-bond distances along with the distance between the sidechains of NK1R-R130^3.50^ (of the DRY microswitch motif) and NK1R-Y305^7.53^.

**Figure 7:**
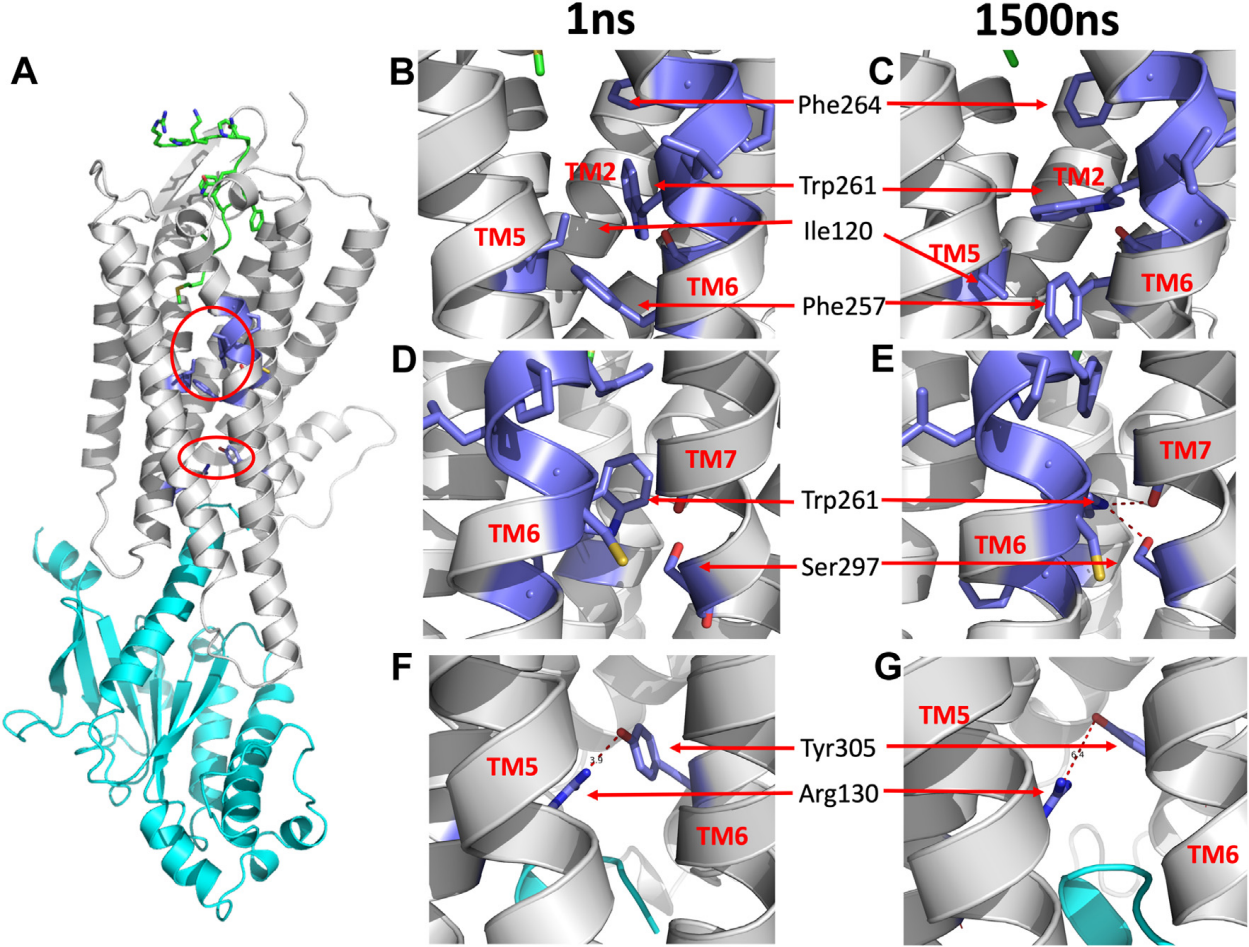
**Conformational changes on activation—snapshots at 1 ns and 1500 ns.***A*, snapshot of NK1R:SP:G_q_ complex at 1 ns. SP is in *green stick model*, NK1R in *gray cartoon*, the sidechains discussed are in *blue*, and G_q_ in *cyan cartoon*. The *upper (lower) oval encircles* the regions highlighted in *B*-*E* (*F*–*G*). The *middle (right) panels* depict structural snapshots at 1 ns (1500 ns). Names of sidechains discussed in the text are shown with *arrows* to guide the eyes. *B* and *C*, sidechains from the PIF motif (NK1R-Ile120^3.40^ and NK1R-Phe257^6.44^) and CWxP (NK1R-Trp261^6.48^) rotated upon activation. *D* and *E*, H-bonds form between the indole moiety of NK1R-Trp261^6.48^ and the sidechain of NK1R-S297^7.45^ (in TM7), as well as with the mainchain carbonyl of NK1R-A294^7.41^ (in TM7), following activation and *F* and *G* interaction between NK1R-Arg130^3.50^ (of the D**R**Y microswitch motif) and NK1R-Tyr305^7.53^ (of the NPxx**Y** microswitch motif) are stabilized following activation. NKR, neurokinin receptor; SP, substance P; TM, transmembrane.

The activation process is further illustrated in Fig. 7. An overview of the NK1R:SP:G_q_ complex is shown in Fig. 7*A* with SP in sticks (green), NK1R (gray), and G_q_ (cyan) cartoon, respectively. The sidechains discussed are shown in sticks (blue). The red ovals encircle regions highlighted in Fig. 7, *B*–*E* (upper oval) and Fig. 7, *F* and *G* (lower oval), respectively. Snapshots from the simulation are shown at 1 ns (middle panels) and 1500 ns (right panels). Fig. 7, *B* and *C* display the conformational changes of the sidechains involved in the concerted motion occurring after 500 ns (Fig. 6). The toggle switch is sandwiched between NK2R-F266^6.51^ and NK1R-F257^6.44^ before and after the concerted motion. The formation of H-bonds between the indole group of the toggle switch tryptophan and the sidechain of NK1R-S297^7.45^ and the mainchain carbonyl of NK1R-A294^7.41^ is depicted in Fig. 7, *D* and *E*. Finally, in panels F and G, the changed interaction between NK1R-R130^3.50^ (of the DRY microswitch motif) and NK1R-Y305^7.53^ (of the NPxxY microswitch motif) is illustrated. To test the robustness of the simulation results, the simulations were repeated at 310 K and 290 K, demonstrating the same qualitative behavior as shown in Fig. 6, *A* and *B*, see Table S2. However, at 310 K, the concerted motion was observed at 900 ns, whereas at 290 K, it occurred at 600 ns.

To explore the impact of SP and G_q_ on the stable, final configuration after 1500 ns of MD simulation (Figs. 6, *A* and *B* and 7, *C*, *E*, and *G*), the structure at 1500 ns was stripped of SP and G_q_ and prepared for continuing simulation (according to the protocol in Experimental procedures). This simulation was run for additional 1500 ns to explore how the fully activated NK1R reverts to its apo structure. Fig. 6, *C* and *D* shows the χ_2_ torsion angles for sidechains and distances corresponding to panels A and B, respectively. In Fig. 6, *A* and *C* concerted rotation of the torsion angles is observed after approximately 100 ns, resulting in the return of the χ_2_ torsion angle of NK1R-W261^6.48^ (in green) to that observed for the first 500 ns in Fig. 6*A*. In Fig. 6*D*, the calculated distances suggest that the two H-bonds described above become intermittent and weakened in the absence of SP and G_q_.

A peculiar characteristic of the interaction where the NK1R-W261^6.48^ indole group forms hydrogen bonds with both NK1R-S297^7.45^ and NK1R-A294^7.41^ is the consistent presence of an unusual and low-probability sidechain configuration characterized by χ_1_ ∼ −65 and χ_2_ ∼0 for the tryptophan (41), as seen in Fig. 6. In the period preceding the concerted rotation, the angles adopt χ_1_ ∼ −65 and χ_2_ ∼120, which present a high-probability rotamer in the *trans* configuration. Even more interesting is the observation from simulation of the NK1R apo structure (Fig. 6, *C* and *D*) that the absence of SP and G_q_ results in a transition of the χ_2_ angle of NK1R-W261^6.48^ from the low-probability back to the high-probability rotameric state. In the MD trajectories by Harris *et al.* (28), the low-probability configuration defined by χ_2_ ∼0 is never observed (Fig. S4). In MD simulations of NK2R:NKA:G_q_ complex, the same peculiar χ_2_ torsion angle is observed.

#### Impact of G protein

The observation of the formation of two H-bonds in the activation process raises the question of the impact of G protein. To that end, the trajectories from Harris *et al.*’s simulations of NK1R:SP and NK1R:SP(6–11) complexes (28) mentioned above were analyzed. In Fig. 5 the distances between the indole of NK1R-W261^6.48^, the NK1R-A294^7.41^ mainchain carbonyl, and a sidechain of NK1R-S297^7.45^ have been calculated and plotted against each other. The results from all 12 trajectories from each complex, shown in different colors, have been superimposed and are depicted in Fig. 5*A* for NK1R:SP and Fig. 5*B* for NK1R:SP(6–11), respectively. Black dots indicate the distances taken from the cryo-EM structures (PDB IDs: 7p00 (27) and 7rmh (28)). Large variations are observed but the results from the two complexes are very similar. Histograms depicting the distance propensities (in the top and to the right of the figures) show intermittent H-bond formation between the indole of NK1R-W261^6.48^ and the sidechain of NK1R-S297^7.45^ but absence of H-bond between indole of NK1R-W261^6.48^ and mainchain carbonyl of NK1R-A294^7.41^. Hence, these results suggest that the presence of the agonist only partially activates the receptor. Full activation of the receptor requires the presence of the G protein to induce the formation of the two H-bonds according to our hypothesis.

#### Elucidating impact of NK1R-S297^7.45^A mutation on activation

The observation of H-bond formation between the toggle switch and the kink of TM7 highlights the significance of S297^7.45^ in the activation process. This is corroborated by the fact that mutating this residue to alanine results in the complete loss of G_s_ activity and a 75% reduction in G_q_ efficacy (15). Since NK1R-S297^7.45^ apparently facilitates the rotation and stabilization of NK1R-W261^6.48^ in the anticipated active conformation, the mutant NK1R-S297^7.45^A was introduced in the model of the NK1R:SP:G_q_ complex and simulated for 1500 ns at 300 K. Interestingly, NK1R-W261^6.48^ and the sidechains discussed above remained stable and did not rotate like in the WT construct. Additionally, no H-bond formation from the indole of NK1R-W261^6.48^ to the mainchain carbonyl of NK1R-A294^7.41^ was observed. This is illustrated in Fig. S5, where the torsion angles (panel A) and the distance between the indole’s NH and the carbonyl of NK1R-A294^7.41^ (panel B) are computed for the MD trajectory. To test the robustness of the simulation results, the simulations were repeated at 310 K and 290 K, demonstrating the same qualitative behavior as shown in Fig. S5, *A* and *B*, see Table S2.

#### Elucidating impact of NK1R-S297^7.45^N mutation on activation

To further investigate the significance of position 297 in NK1R, sequence variability among human GPCRs with peptide agonists was analyzed in GPCRdb (42). Thus, alignment revealed that only three residues (Ser, Asn, and Gly) are found in that position, with Asn present in 57 receptors (75%), Ser in 18 (24%) and Gly in one GPCR. Given the high prevalence of Asn, we introduced S297^7.45^N mutation into the NK1R:SP:G_q_ complex and simulated for 1500 ns at 300 K. The resulting trajectory analysis (Fig. S5, *C* and *D*) showed stable torsion angles (panel C) and no H-bonds between the indole of NK1R-W261^6.48^ and the TM7 kink (panel D). To test the robustness of the simulation results, the simulations were repeated at 310 K and 290 K, demonstrating the same qualitative behavior as shown in Fig. S5, *C* and *D*, see Table S2. Hence, according to the hypothesized activation mechanism, the NK1R-S297^7.45^N mutant should be inactive. This was confirmed through activation assays (BRET-based cAMP and IP_3_ accumulation assays, results in Fig. S6) where the mutant NK1R-S297^7.45^N activated by NKA was found to have considerably lower affinity and efficacy, while in NK2R-S299^7.45^N activated by NKA, the activity was absent.

### Elucidating impact of NK1R-N50^1.50^A mutation on activation

Another mutant, NK1R-N50^1.50^A, experienced a complete loss of activity (15). In the structure of NK1R:SP:Gq (PDB ID: 7p00), the sidechain of NK1R-N50^1.50^ H-bonds to the sidechain of NK1R-T298^7.46^ (both conserved among NKRs), potentially stabilizing the kinked conformation of TM7. The mutant NK1R-N50^1.50^A was introduced in the model of the NK1R:SP:G_q_ complex and simulated for 1500 ns at 300 K (Fig. S5, *E* and *F*). The torsion angles for the sidechains mentioned above are shown as a function of time (panel E). After a few nanoseconds of simulation, the sidechains adopt the anticipated active conformations including H-bonds to the kink of TM7 (panel F). However, after 500 nanoseconds, the sidechains collectively swung back to the orientation observed in Fig. S5*E* (and remained there for the duration of the simulation), leading to the loss of direct H-bond interactions with TM7 (Fig. S5*E*). The robustness of the simulation results were tested by repeating the simulations at 310 K and 290 K, demonstrating the same qualitative behavior as shown in Fig. S5, *E* and *F*, see Table S2. Therefore, mutagenesis studies as well as MD simulations support the detailed aspects of the activation mechanism as outlined. Notably, Asn is conserved across all human GPCRs with peptide ligands (42). The role of NK1R-S297^7.45^ is that of a central hub that establishes connections between the orthosteric site and the G protein–binding region.

The outcome of analyses of MD trajectories suggests a dynamic interplay between certain sidechains of the CWxP and PIF microswitch motifs, which results in H-bond interaction from the tryptophan’s indole to H-bond acceptors in the kink of TM7. In complex with the endogenous ligand, partial H-bond generation is observed from the toggle switch indole to the central hub, as illustrated in Fig. 5. The sidechain of the toggle switch NK1R-W261^6.48^ adopts a high-probability configuration, characterized by χ_2_ ∼90 (Fig. S4). When complexed with G protein, the toggle switch then transitions to a low-probability configuration, characterized by χ_2_ ∼0, allowing the indole group to form an additional H-bond with the mainchain carbonyl of NK1R-A294^7.41^. This facilitates the maturation of the interface to the G protein. Interestingly, a similar activation mechanism was proposed for the class B glucagon receptor (43), where computational studies demonstrated that glucagon stabilizes the receptor in a preactivated complex. Full activation of the receptor occurs only upon G protein binding, in a mechanism consistent with available cryo-EM data (43).

The notion of a partially activated receptor in the presence of a ligand, according to the present hypothesis, could explain the observed very low ligand-independent activity (*i.e.,* no constitutive activity). The proposed multistate mechanism suggests that a spontaneous transition to the active state is unlikely. Therefore, the observed presence of (naively) low-probability configurations for the toggle switch NK1R-W261^6.48^ indicates that the typical high-probability rotameric state is associated with the partially activated receptor, whereas the fully activated receptor necessitates the toggle switch to adopt the low-probability state that maximizes H-bonds in the microenvironment (to NK1R-A294^7.41^ and NK1R-S297^7.45^).

## Conclusion

The tachykinin family of receptors has long served as a valuable model system in neuropeptide research. Many of the discoveries in this field have led to significant advancements, including a wealth of structural information particularly on NK1R and more recently a rekindled interest in NK2R. Our findings expose the critical role of specific residues at the bottom of the orthosteric site in affecting NK1 and NK2 receptor activation by neuropeptides SP and NKA. Notably, the mutation NK1R-F264^6.51^Y significantly influences NKA activation, while changes in SP activation are comparatively modest. Interestingly, the significant increases in potency of NK1R mutations in the β-arrestin recruitment assay following activation by NKA closely parallel those observed in the BRET-based cAMP assay, demonstrating a consistent ranking in potency. In contrast, when the mutants are activated by SP, only a slight increase in efficacy is observed, with no improvement in potency. The distinct activation patterns of the NK1R mutants when stimulated by NKA, as shown in Fig. 3, *A*, *C* and *E*, indicate a signaling bias toward Gs and β-arrestin pathways. Considering the structural information presented in the introduction, this outcome is somewhat unexpected (although it has been observed previously (15, 28)). Fully understanding this phenomenon would require an in-depth pharmacological, biophysical, and computational study, which is beyond the scope of the current work. These observations underscore the importance of the two mutated residues at the bottom of the orthosteric site in drug design processes, where selectivity and biased signaling are crucially important.

MD simulations strongly suggest that the microswitch tryptophan indole group plays a crucial role, forming hydrogen bonds progressively: initially unproductive when inactivated, partially upon neuropeptide binding, and fully with both NK1R-S297^7.45^ and NK1R-A294^7.41^ upon both neuropeptide and G protein binding. The partially activated receptor was seen in MD trajectories from MD simulations of NK1R:SP and NK1R:SP (6–11) complexes, which revealed the presence of only an intermittent H-bond between the toggle switch tryptophan and the hub. This contrasted with the observation of two stable H-bonds between the toggle switch tryptophan and the kink of TM7 when the G protein is present. In the fully activated receptor, the χ_2_ torsion angle of the toggle switch NK1R-W261^6.48^ populates a low-probability sidechain configuration while in simulations of the NK1R apo structure, NK1R:SP (and NK1R:SP6–11), and mutated complexes, a high-probability sidechain configuration is observed. The mutants NK1R-S297^7.45^A, NK1R-S297^7.45^N (and NK2R-S299^7.45^N) and NK1R-N50^1.50^A support the activation hypothesis because they exhibit little to no activity upon stimulation with SP. We, therefore, conclude that the dual H-bond formation from the toggle switch tryptophan indole group to the H-bond acceptors in the kink of TM7 facilitates signaling to NPxxY microswitch motif and the G protein–binding interface. The high structural and sequence similarity (Fig. S1) of NKRs supports the notion that they are activated in the same way. Finally, we speculate that the notion of a partially activated receptor in the presence of a ligand could explain the very low observed ligand-independent activity of the receptor (*i.e.*, lack of constitutive activity) because the proposed multiple-state mechanism suggests that a spontaneous transition to the active state is unlikely.

## Experimental procedures

### Structural modeling

Structural visualization was performed in PyMOL (open_source version 2.5.0, Schrödinger LLC, http://www.pymol.org/pymol). The mutants NK1R-F264^6.51^Y, NK1R-S297^7.45^A, and NK1R-N50^1.50^A were introduced in the cryo-EM structure of NK1R (PDB ID: 7p00 (44)) in PyMOL. Structural analyses were performed in Maestro (Schrödinger Release 2024-2: Maestro, Schrödinger, LLC, 2024.)

### MD simulations

*Desmond/OPLS force field*: The NK1R:SP:Gq model (PDB ID: 7p00 (27)) in combination with the publicly available IPython notebooks to access ColabFold/AlphaFold2 was prepared (45, 46, 47) for simulation in Desmond/OPLS (37). The Desmond simulations simulated in the NPgT ensemble at 310 K, 1 bar applying a surface tension of 4000 bar Å. The Desmond minimization protocol uses the steepest descent method. The protocol includes restraint followed by nonrestrained equilibration steps, each running for 5 ns. During the equilibration process, the cut-off for van der Waals and short-range electrostatic interactions was set to 9 Å. Reversible Reference System Propagator Algorithm integrator was used with a time step of 2 fs, and long-range electrostatics were computed every 6 fs. The system was equilibrated in the NVT ensemble for 100 ps using Brownian dynamics with T = 10 K and 50 kcal/mol/Å^2^ restrains applied on solute heavy atoms. This was followed by a Brownian equilibration in the NPT ensemble at T = 50 K and a H_2_O barrier and restraints (force constant = 5 kcal/mol/Å^2^.) applied on the membrane in the z-direction and the protein. Finally, the system was equilibrated in the NPgT ensemble at T = 50K using the same restraints, followed by gradual heating from 100 K to 300 K, using a H_2_O barrier and gradual release of the restrains. This was followed by two NVT production steps with all restraints removed. The final production simulations were performed after a brief minimization. For the simulation, the customized OPLSe3 force field was used (48). As a housekeeping metric, the first 50 ns of the simulations were not included in the analysis. Plotting was done using Grace (xmgrace; https://plasma-gate.weizmann.ac.il/Grace/) or matplotlib (49).

### Compounds

All compounds were dissolved in 100% dimethyl sulfoxide or 70% ethanol. NKA and SP were from Sigma-Aldrich (N4267 and S6883, respectively).

### Plasmids

All receptor constructs of wildtype and modified human *TACR1* and *TACR2* were inserted into the pcDNA3.1(+)-C-DYK vector (GenScript), whereas CAMYEL (20) was expressed *via* pcDNA3.1(+) vector.

The membrane-targeted citrine-SH3 and Renilla luciferase (Rluc) 8-Arr2-SP1 fusion protein construct were both expressed in pcDNA3.1(+) vector (50).

### Cell culture, plating, and transfection

COS7 cells were maintained in Dulbecco’s Modified Eagle’s Medium 1885 with GlutaMAX supplemented with 10% fetal bovine serum (Sigma-Aldrich), 100 units/ml penicillin, and 100 μg/ml streptomycin at 37 °C with 10% CO_2_. COS7 cells were tested monthly for *mycoplasma* and kept negative. COS7 cells were plated in either clear, white, or white with clear bottom 96-well plates that were coated with poly-D-lysine (Sigma-Aldrich) 30 min prior to cell seeding (20,000 cells/well). The following day plates were transiently transfected using calcium precipitation with 200 ng/well DNA in a culture medium with the addition of 100μM (final concentration) chloroquine. Transfection was stopped 5 h later with the addition of a fresh maintenance medium. Fig. S4 illustrates the consistency in expression levels as determined through ELISA assay for both WT receptors and mutants, demonstrating robust reproducibility.

### BRET-based cAMP assay

The intracellular level of cAMP was monitored in real-time using BRET. This was achieved by implementing a construct consisting of a cAMP binding protein (exchange protein activated by cAMP [EPAC]) which has been flanked by a BRET pair consisting of Rluc and yellow fluorescent protein. This construct is called CAMYEL (cAMP sensor using yellow fluorescent protein-EPAC-Rluc) and enables cAMP production to be sensed as EPAC changes conformation in response to increasing levels of cAMP, ultimately resulting in a loss of BRET signal.

On the day of the assay, white 96-well plates with COS7 cells were washed twice with 100 μl/well Hank's Balanced Salt Solution (HBSS) (GIBCO, Life Technologies) and preincubated for 30 min at 37 °C with 85 μl HBSS. Luciferase substrate coelenterazine h (Thermo Fisher Scientific) was added and a baseline measurement was taken after 5 min. Dose-response curves of either NKA or SP were added and measurements were recorded every minute for 30 min on a CLARIOstar Plus plate reader. The BRET signal was calculated as the ratio of emission intensity at 535 nm (citrine) to the emission intensity at 475 nm (luciferase). Determinations were made in triplicates.

### IP_3_ accumulation assay

COS7 cells seeded in clear 96-well plates were incubated with 0.5 μCi/ml myo [3H]inositol (Perkin Elmer) in 100 μl growth medium overnight following the transfection. The subsequent day cells were washed twice with 200 μl/well HBSS (GIBCO, Life Technologies) and preincubated for 5 min at 37 °C with 100 μl/well HBSS buffer supplemented with 10 mM LiCl. Ligand addition was followed by 150 min incubation at 37 °C. To stop ligand incubation, cells were lysed with 40 μl 10 mM formic acid followed by 30 min incubation on ice. Thirty five microliters of the lysate was transferred to white 96-well plates together with 60 μl of 1:8 diluted YSi SPA scintillation beads (Perkin Elmer). Plates were sealed and vigorously shaken for 15 min, followed by 5 min centrifugation at 1500 rpm. Measurements (scintillation) were recorded on a Microbeta (PerkinElmer) after a 4 h delay and determinations were made in duplicates.

### BRET-based arrestin recruitment assay

The recruitment of arrestin-2 was assessed using BRET. A membrane-targeted citrine-SH3 construct was coexpressed with RLuc8-Arr2-SP1 fusion protein to monitor the membrane recruitment of arrestin following GPCR activation. The inclusion of SH3 and SP1 domains enhances the interaction of the complexes, facilitating more efficient energy transfer and yielding a stronger BRET signal. This interaction occurs exclusively when arrestin is actively recruited to the membrane.

On the day of the assay, cells were washed twice with 100 μl of HBSS (GIBCO, Life Technologies) and preincubated with 85 μl of HBSS for 30 min at 37 °C. Baseline measurements were taken 5 min after the addition of the luciferase substrate coelenterazine H (Thermo Fisher Scientific). Dose-response curves of either NKA or SP were added, and measurements were recorded every 2 min for 20 min on a CLARIOstar Plus plate reader. The BRET signal was calculated as the ratio of emission intensity at 535 nm (citrine) to the emission intensity at 475 nm (luciferase). Determinations were made in triplicates.

### Statistical analysis

All dose-response curves have been calculated using Prism 10 software (GraphPad Software, www.graphpad.com) nonlinear regression with four parameters. All data are shown as mean ± SEM unless stated otherwise and consist of two technical replicates from three biological replicates. In Table S1 the EC_50_ and E_max_ values from functional assays are tabulated.

## Data availability

MD trajectories corresponding to Figs. 2, 6, 7, S2, and S3 have been uploaded (https://doi.org/10.5281/zenodo.1207250) and an overview of simulation temperatures and simulation lengths is provided in Table S2.

## Supporting information

This article contains supporting information.

## Conflict of interests

The authors declare that they have no conflicts of interest with the contents of this article.
