# Supplementary material for: Molecular determinants of neuropeptide-mediated activation mechanisms in tachykinin NK1 and NK2 receptors
Source: J Biol Chem. 2024 Oct 30;300(12):107948. doi: 10.1016/j.jbc.2024.107948 (PMC11625327; doi:10.1016/j.jbc.2024.107948)
Supplement: Supporting information [file mmc1.docx]

**Supporting Information**

**Molecular determinants of neuropeptide-mediated activation mechanisms in tachykinin NK1 and NK2 receptors**

Jacob E. Petersen^1^, Artem Pavlovskyi^1^, Jesper J. Madsen^2,3^, Thue W. Schwartz^1^, Thomas M. Frimurer^1^, and Ole H. Olsen^1*^

^1^ Section for Metabolic Receptology, Novo Nordisk Foundation Center for Basic Metabolic Research, University of Copenhagen, Blegdamsvej 3b, DK-2200 Copenhagen N, Denmark

^2^ Department of Molecular Medicine, Morsani College of Medicine, University of South Florida, Tampa, Florida 33612, United States of America

^3^ Center for Global Health and Infectious Diseases Research, Global and Planetary Health, College of Public Health, University of South Florida, Tampa, Florida 33612, United States of America

^*^Correspondence: Ole H. Olsen, Section for Metabolic Receptology, Novo Nordisk Foundation Center for Basic Metabolic Research, University of Copenhagen, Blegdamsvej 3b, DK-2200 Copenhagen N, Denmark. Tel.: +45 60661288. E-mail: [oho@sund.ku.dk](mailto:oho@sund.ku.dk)

This document contains six figures (Figs. S1-6) and two tables (Table S1-2)


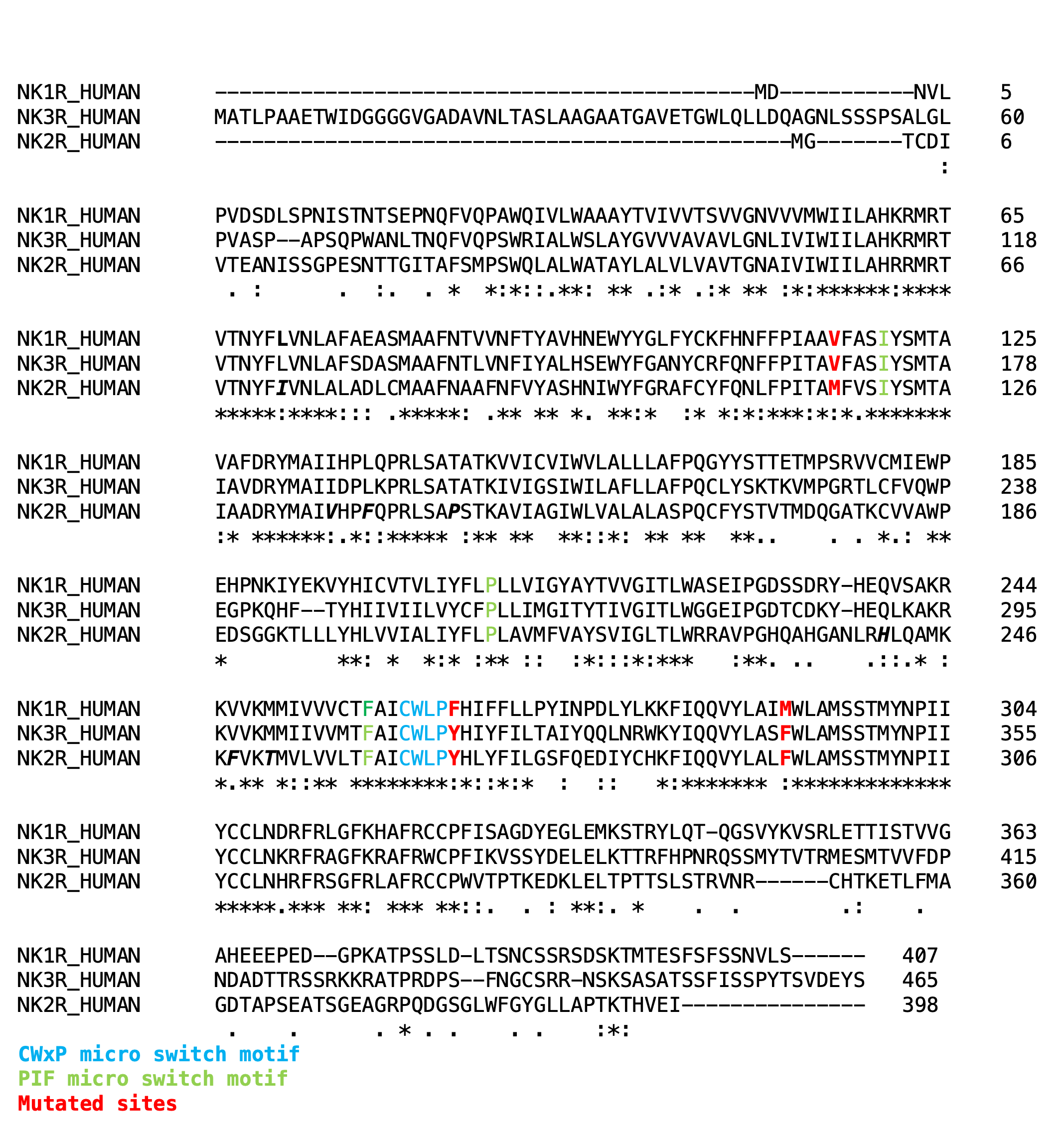


**Figure S1: Sequence alignment of human NKRs** (Clustal W (49)). The microswitch motifs discussed in the introduction in blue and green. The mutated sites NK1R-F264^6.51^Y, NK1R-M291^6.51^F and NK1R-V116^3.36^M in red


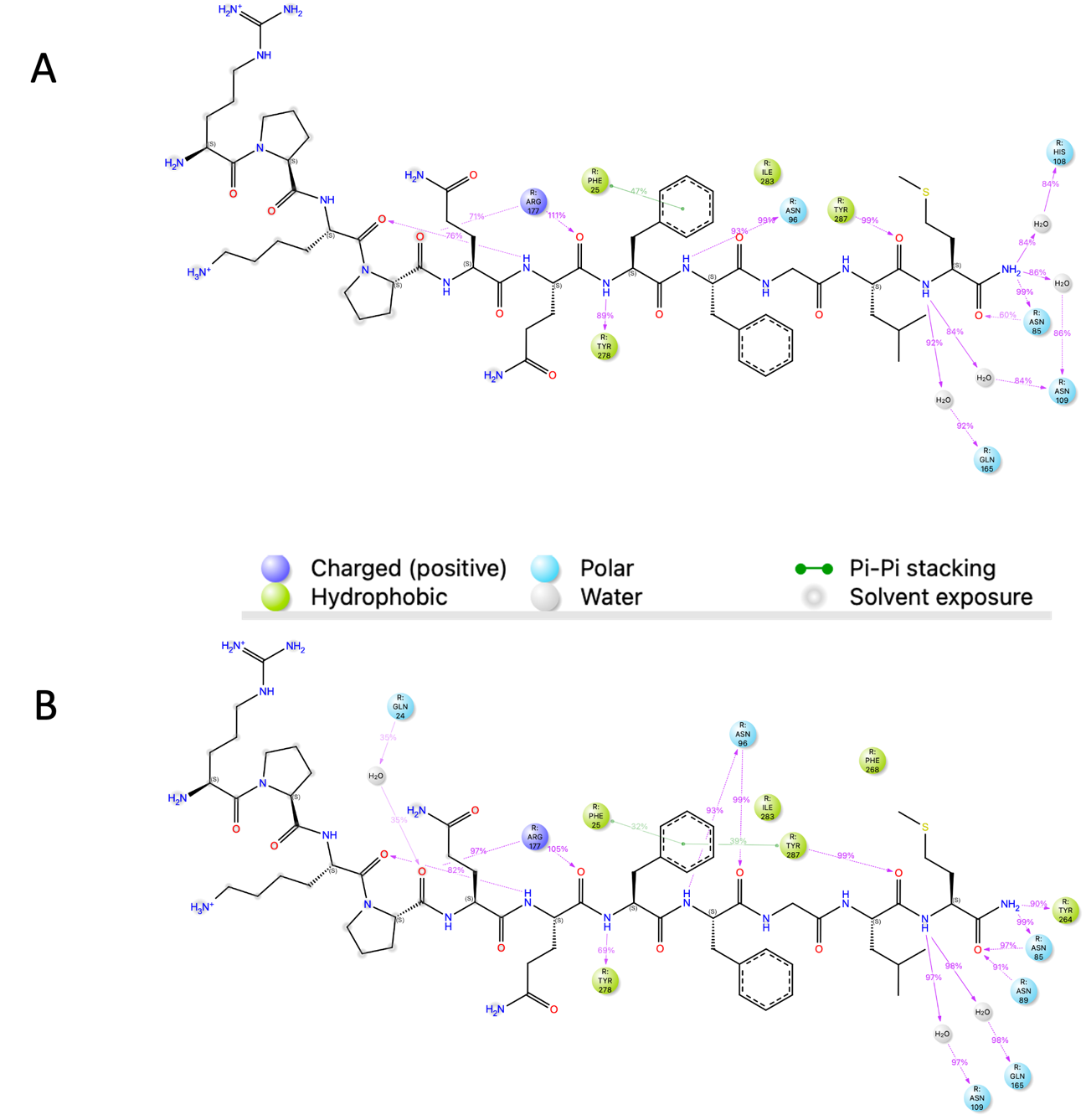


**Figure S2. Interaction diagrams for full-length SP (RPKPQQFFGLM-NH_2_) and NK1R and NK1R-F264^6.51^Y receptors generated from MD trajectories (1500 ns, at 300K).** Features appearing in the diagrams and what they represent are shown between the diagrams. Interactions between SP1-9 and the receptors are virtually identical while the C-terminal interactions differ induced by the introduction of mutation F264^6.51^Y.


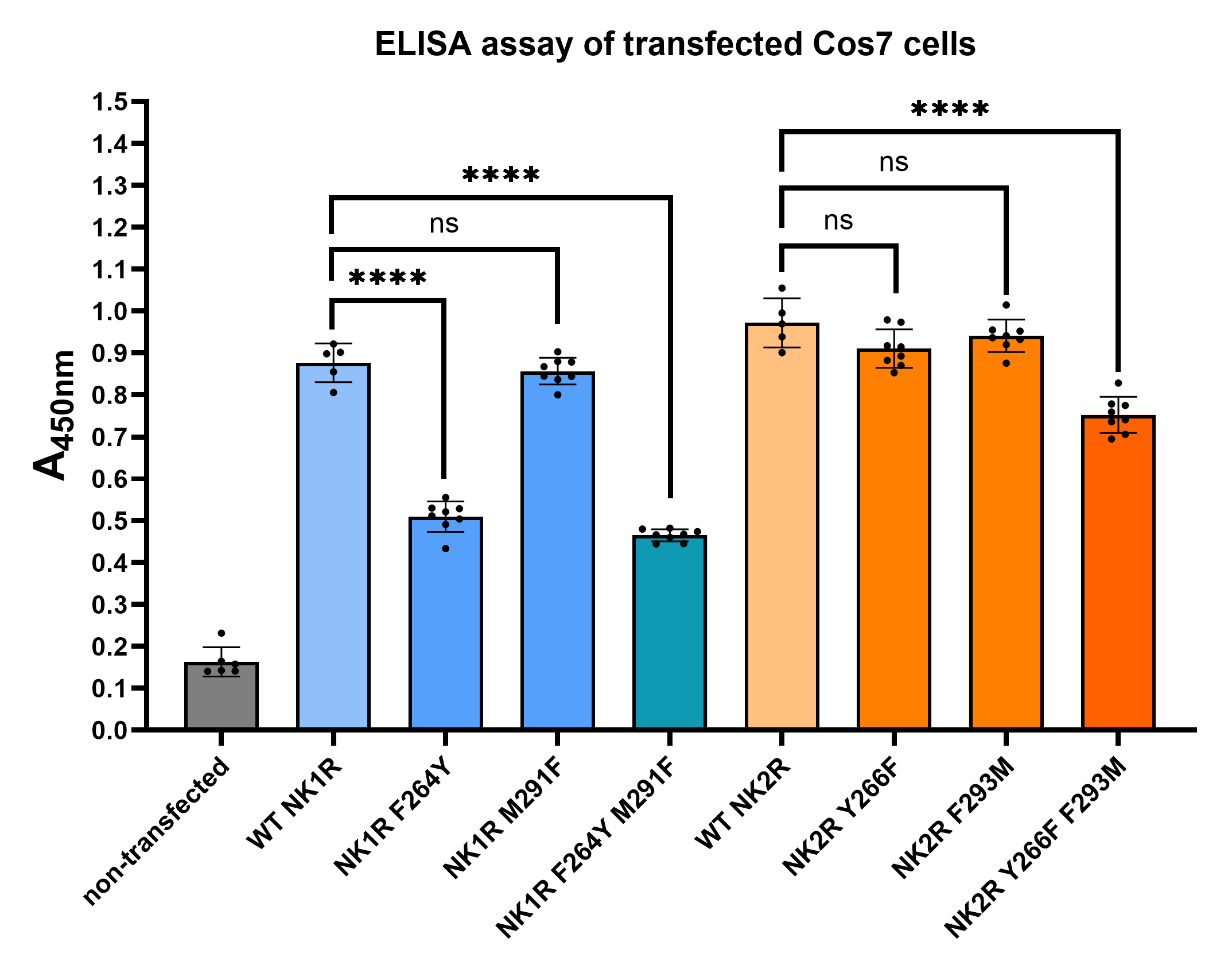
**Figure S3. Elisa assay data.** Consistency in expression levels as determined through Elisa assay for both wildtype receptors and mutants, demonstrating robust reproducibility. The expression levels of NK1R mutants containing F264^6.51^Y are approximately 50% of that of NK1R.


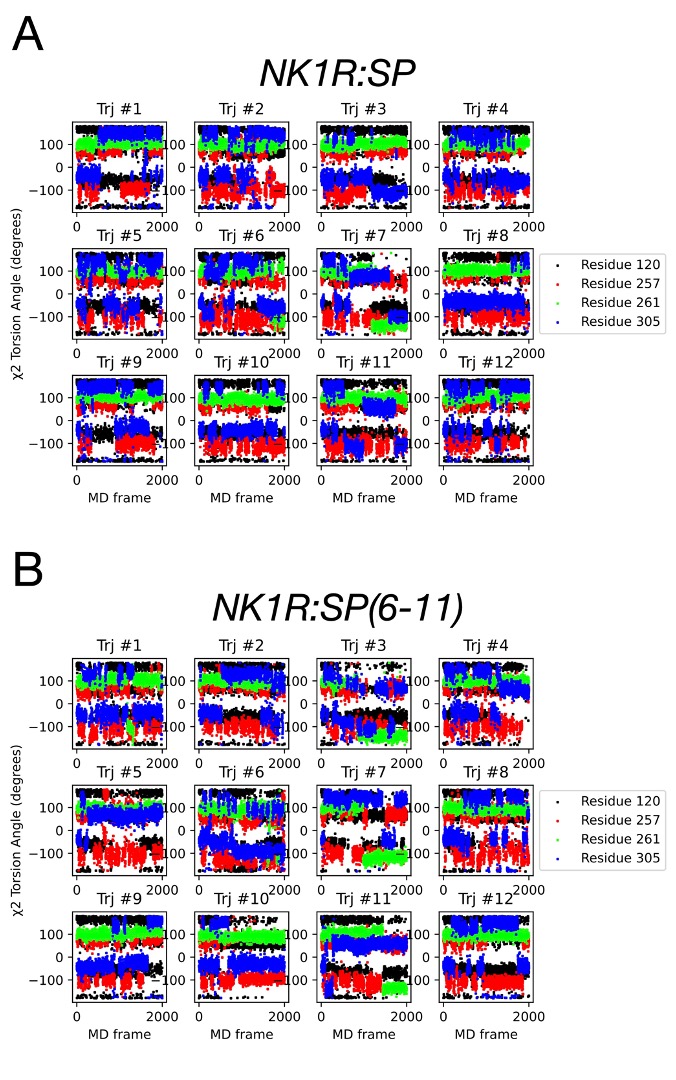


**Figure S4.** **Analyses of simulations of NK1R:SP and NK1R:SP(6-11) complexes.** χ_2_ torsion angles as a function of time for selected sidechains of 12 trajectories of each complex taken from (28). It is of note that χ_2_ for NK1R-W261^6.48^ (green) never approaches zero. Only high-probability rotameric states are observed.


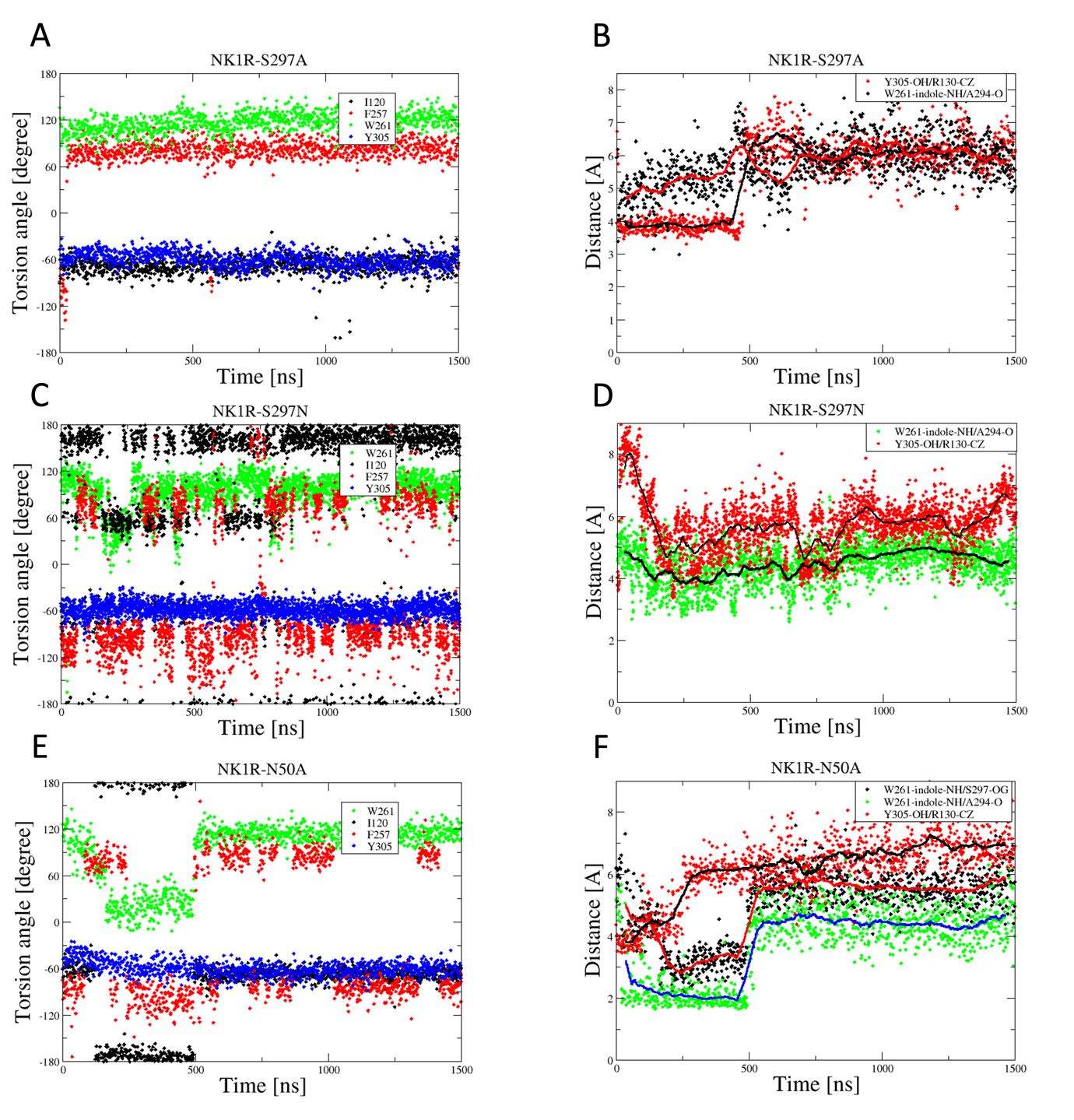


**Figure S5. Analyses of trajectories from MD simulations of NK1R:SP:G_q_ complex with the mutations incorporated at** **300 K.** (A), (B) MD simulations of NK1R-S297^7.45^A mutation in NK1R:SP:G_q_ complex. (A) χ_2_ torsion angles as a function of time for selected sidechains of microswitch motifs: NK1R-I120^3.40^ and NK1R-F257^6.44^ (P**IF** motif, red), NK1R-W261^6.48^ (C**W**xP motif, green) and NK1R-Y305^7.53^ (NPxx**Y**, black). The sidechain conformations are stable, and no rotations are observed. (B) Distance between the indole group of NK1R-W261^6.48^ and the mainchain carbonyl of NK1R-A294^7.41^ (depicted in black). The distance between the sidechains of NK1R-R130^3.50^ (of the D**R**Y microswitch motif) and NK1R-Y305^7.53^ (of the NPxx**Y** microswitch motif) is in red. (C), (D) MD simulations of NK1R-S297^7.45^N mutation in NK1R:SP:G_q_ complex. χ_2_ torsion angles as a function of time for selected sidechains of microswitch motifs as described for (A). (C) stable torsion angles , (D) the Hbonds to the kink of TM7 are missing. (E), (F) MD simulations of NK1R-N50^1.50^A mutation in NK1R:SP:G_q_ complex. (E) χ_2_ torsion angles as a function of time for selected sidechains of microswitch motifs as described for (A). After 500 ns the torsion angles are stabilized as in (A) apart from NK1R-F257^6.44^ which flips between two states. In (F) it is shown how Hbonds between the indole of NK1R-W261^6.48^ and the kink of TM7 are abrogated. Running averages have been shown in (B), (D) and (F) to guide the eyes.


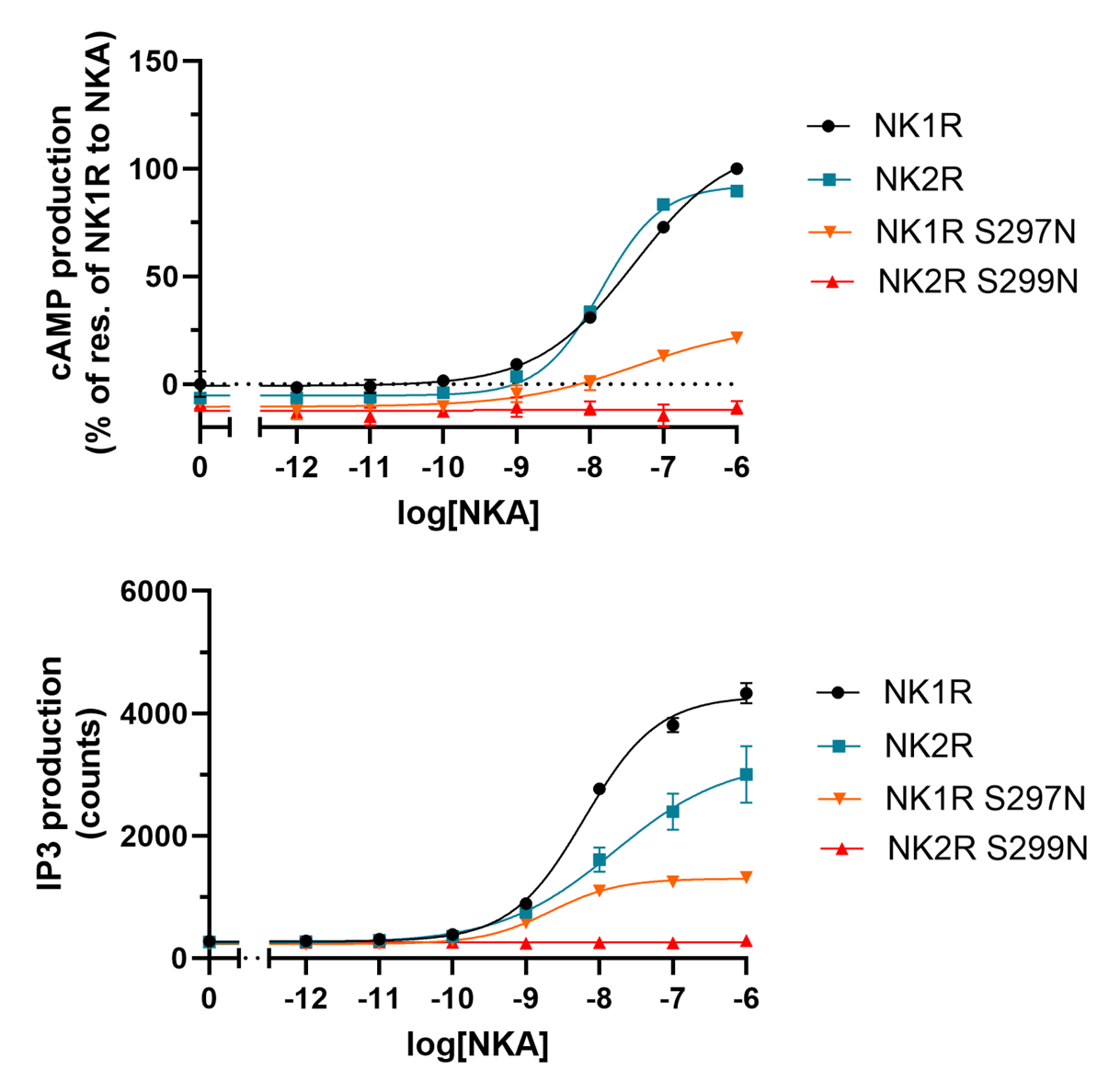


**Figure S6. Activation of NK1R-S297^7.45^N mutant by NKA.** Mutant tested in BRET-based cAMP assay (upper panel) and in IP_3_ accumulation assay (lower panel). Lowered affinity and efficacy in NK1R-S297^7.45^N and no activation in NK2R-S299^7.45^N. All data are shown as mean ± SEM of three independent experiments, each run with techical triplicates.

**Table S1A, activity data for figure 3**

| Assay type | Receptor | Ligand | log EC_50_ | E_max_ [% of corresponding NKR WT activity] | Hill slope |
| --- | --- | --- | --- | --- | --- |
| cAMP | NK1R WT | NKA | -7,78 ± 0,09 | 100 ± 2,6 | 0,63 |
|  | NK1R F264Y |  | -8,33 ± 0,10 | 121,6 ± 5,8 | 0,76 |
|  | NK1R M291F |  | -7,97 ± 0,08 | 99,9 ± 3,1 | 0,67 |
|  | NK1R F264Y M291F |  | -8,50 ± 0,11 | 124,9 ± 6,7 | 0,91 |
|  | NK1R WT | SP | -9,11 ± 0,07 | 100 ± 0,1 | 1,528 |
|  | NK1R F264Y |  | -9,16 ± 0,13 | 116,7 ± 5,3 | 1,421 |
|  | NK1R M291F |  | -9,12 ± 0,09 | 98,7 ± 2,2 | 1,348 |
|  | NK1R F264Y M291F |  | -9,18 ± 0,14 | 111,6 ± 5,0 | 1,687 |
| IP3 | NK1R WT | NKA | -8,32 ± 0,06 | 100 ± 0.1 | 0,88 |
|  | NK1R F264Y |  | -8,44 ± 0,12 | 105 ± 6,3 | 0,99 |
|  | NK1R M291F |  | -8,49 ± 0,23 | 102 ± 12,7 | 1,02 |
|  | NK1R F264Y M291F |  | -8,53 ± 0,19 | 102 ± 11,0 | 1,02 |
|  | NK1R WT | SP | -8,46 ± 0,10 | 100 ± 7,0 | 1,17 |
|  | NK1R F264Y |  | -8,71 ± 0,18 | 91 ± 5,4 | 1,71 |
|  | NK1R M291F |  | -8,64 ± 0,35 | 97 ± 17,4 | 1,53 |
|  | NK1R F264Y M291F |  | -8,73 ± 0,27 | 98,5 ± 11,1 | 1,64 |

**Table S1B, activity data for figure 4**

| Assay type | Receptor | Ligand | log EC_50_ | E_max_ [% of corresponding NKR WT activity] | Hill slope |
| --- | --- | --- | --- | --- | --- |
| cAMP | NK2R WT | NKA | -8,33 ± 0,18 | 100 ± 2,6 | 1,02 |
|  | NK2R Y266F |  | -8,36 ± 0,32 | 60,4 ± 6,8 | 1,20 |
|  | NK2R F293M |  | -8,18 ± 0,25 | 88,5 ± 4,8 | 1,06 |
|  | NK2R Y266F F293M |  | N/A | 21 ± 3,5 | N/A |
|  | NK2R WT | SP | N/A | N/A | N/A |
|  | NK2R Y266F |  | N/A | N/A | N/A |
|  | NK2R F293M |  | N/A | N/A | N/A |
|  | NK2R Y266F F293M |  | N/A | N/A | N/A |
| IP3 | NK2R WT | NKA | -8,09 ± 0,09 | 100 ± 0,1 | 0,71 |
|  | NK2R Y266F |  | -8,74 ± 0,39 | 32,5 ± 7,0 | 0,57 |
|  | NK2R F293M |  | -8,18 ± 0,48 | 107,6 ± 20,0 | 0,62 |
|  | NK2R Y266F F293M |  | -8,47 ± 0,42 | 48 ± 9,9 | 0,58 |
|  | NK2R WT | SP | N/A | N/A | N/A |
|  | NK2R Y266F |  | N/A | N/A | N/A |
|  | NK2R F293M |  | N/A | N/A | N/A |
|  | NK2R Y266F F293M |  | N/A | N/A | N/A |

**Table S1.** EC_50_ and E_max_ values from functional assays corresponding to data in Figures 3 and 4.

**Table S2. MD simulation details**

| **Complexes, simulation details** | **Reference to figures** |  |
| --- | --- | --- |
| **NK1R:SP**, 1500ns, 300 K | Figures 2 and S2 |  |
| **NK1R-F264^6.51^Y:SP**, 1500ns, 300 K | Figures 2 and S2 |  |
|  |  |  |
|  | **Activation mechanism** | **Hbond formation to TM7 kink** |
| **NK1R:wt:SP:G_q_** |  |  |
| 290K, 1500 ns | Activated as in Figures 6A,B | Hbond formation at Time =~600ns |
| 300K,1500 ns | See Figure 6A,B | Hbond formation at Time=~500ns |
| 310K,1500 ns | Activated as in Figures 6A,B | Hbond formation at Time =~900ns |
| **NK1R:wt:G_q_** |  |  |
| 300 K,1500 ns | Apo structure, Figures 6C,D | No Hbond formation |
| **NK1R-S297^7.45^A:SP:G_q_** |  |  |
| 290K, 1500 ns | No activation | Weak, intermittent Hbond to A294O |
| 300K, 1500 ns | See Figures S5A,B | No Hbond to TM7 kink |
| 310K, 1500 ns | No activation as in Figures S5A,B | No Hbond to TM7 kink |
| **NK1R-S297^7.45^N:SP:G_q_** |  |  |
| 290K, 1500 ns | Similar to Figures S5C,D | No Hbonds to TM7 kink |
| 300K, 1500 ns | See Figures S5C,D | No Hbonds to TM7 kink |
| 310K, 1500 ns | Similar to Figures S5C,D | No Hbonds to TM7 kink |
| **NK1R-N50^1.50^A:SP:G_q_** |  |  |
| 290K, 1500 ns | Similar to Figures S5E,F | Weak, intermittent Hbonds to TM7 kink |
| 300K, 1500 ns | See Figures S5E,F | No Hbonds to TM7 kink |
| 310K, 1500 ns | Similar to Figures S5E,F | Weak, intermittent Hbonds to TM7 kink |

**Table S2.** Simulation details for trajectories discussed in the text.
